# STING activation in TET2-mutated hematopoietic stem/progenitor cells contributes to the increased self-renewal and neoplastic transformation

**DOI:** 10.1038/s41375-023-02055-z

**Published:** 2023-10-10

**Authors:** Jiaying Xie, Mengyao Sheng, Shaoqin Rong, Dan Zhou, Chao Wang, Wanling Wu, Jingru Huang, Yue Sun, Yin Wang, Pingyue Chen, Yushuang Wu, Yuanxian Wang, Lan Wang, Bo O. Zhou, Xinxin Huang, Colum P. Walsh, Stefan K. Bohlander, Jian Huang, Xiaoqin Wang, Guo-Liang Xu, Hai Gao, Yuheng Shi

**Affiliations:** 1 Institutes of Biomedical Sciences, Shanghai Xuhui Central Hospital, Medical College of Fudan University, Chinese Academy of Medical Sciences (RU069), Shanghai, 200032 China; 2 Center for Medical Research and Innovation, Shanghai Pudong Hospital, Institutes of Biomedical Sciences, Medical College of Fudan University, Shanghai, 201399 China; 3 China State Key Laboratory of Molecular Biology, Institute of Biochemistry and Cell Biology, Center for Excellence in Molecular Cell Science, Chinese Academy of Sciences, Shanghai, 200031 China; 4 Department of Hematology, Huashan Hospital, Fudan University, Shanghai, 200024 China; 5 CAS Key Laboratory of Tissue Microenvironment and Tumor, Shanghai Institute of Nutrition and Health, Chinese Academy of Sciences, Shanghai, 200031 China; 6 Genomic Medicine Research Group, Biomedical Sciences, Ulster University, Coleraine, BT52 1SA UK; 7 Centre for Research and Development, Region Gävleborg/Uppsala University, Gävle, Sweden; 8 Leukaemia & Blood Cancer Research Unit, Department of Molecular Medicine and Pathology, The University of Auckland, Auckland, New Zealand; 9 Coriell Institute for Medical Research, Camden, NJ 08103 USA; 10 Temple University Lewis Katz School of Medicine, Center for Metabolic Disease Research, Philadelphia, PA 19140 USA; 11 Shanghai Key Laboratory of Clinical Geriatric Medicine, Shanghai, Huadong Hospital, Shanghai, 200040 China

**Keywords:** Cancer prevention, Acute myeloid leukaemia

## Abstract

Somatic loss-of-function mutations of the dioxygenase Ten-eleven translocation-2 (TET2) occur frequently in individuals with clonal hematopoiesis (CH) and acute myeloid leukemia (AML). These common hematopoietic disorders can be recapitulated in mouse models. However, the underlying mechanisms by which the deficiency in TET2 promotes these disorders remain unclear. Here we show that the cyclic guanosine monophosphate-adenosine monophosphate synthase (cGAS)-stimulator of interferon genes (STING) pathway is activated to mediate the effect of TET2 deficiency in dysregulated hematopoiesis in mouse models. DNA damage arising in *Tet2*-deficient hematopoietic stem/progenitor cells (HSPCs) leads to activation of the cGAS-STING pathway which in turn promotes the enhanced self-renewal and development of CH. Notably, both pharmacological inhibition and genetic deletion of STING suppresses *Tet2* mutation-induced aberrant hematopoiesis. In patient-derived xenograft (PDX) models, STING inhibition specifically attenuates the proliferation of leukemia cells from TET2-mutated individuals. These observations suggest that the development of CH associated with TET2 mutations is powered through chronic inflammation dependent on the activated cGAS-STING pathway and that STING may represent a potential target for intervention of relevant hematopoietic diseases.

## Introduction

Ten-eleven translocation-2 (TET2) is a dioxygenase that catalyzes the three steps of oxidation of methylated cytosines to facilitate demethylation of genomic DNA [1]. As an epigenetic regulator, TET2 plays important roles in various physiological and pathological processes involving cell fate determination and cancer development. It is essential for the homeostasis of hematopoietic stem/progenitor cells (HSPCs) and in the hematopoietic hierarchy and loss-of-function mutations of *TET2* are prominent drivers of age-related clonal hematopoiesis (CH) in humans [2]. Importantly, large cohort sequencing data suggest that individuals with *TET2* mutations are also at a high risk of developing hematologic malignancies, such as myelodysplastic neoplasms, myeloproliferative neoplasms (MPN) and acute myeloid leukemia (AML) [3–6]. *TET2* loss-of-heterozygosity and somatic mutations serve as driver mutations in nearly 10% of patients with AML [7] and in 17% of individuals with CH [8].

Consistent with clinical observations, mice deficient for *Tet2* show myeloid transformation. Genetic inactivation of *Tet2* in the mouse hematopoietic system disturbed the homeostasis of HSPCs and resulted in aberrant myeloid maturation [9–12]. In particular, *Tet2* deletion altered the HSC compartment by inducing expansion of pre-leukemic lineage-Sca1^+^cKit^+^(LSK) cells and changed their differentiation potential by skewing it toward monocytic/granulocytic lineages [10]. Loss of *Tet2* in HSPCs significantly increased their replating capacity in vitro and self-renewal in competitive bone marrow transplantation (cBMT) assays [11]. However, unlike aggressive AML models induced by for example *KMT2A* fusion genes [13], *Tet2*-deficient mice showed a long latency before developing leukemia [10, 11]. Genetic studies of AML patients indicated that mutations in *TET2* were often acquired as one of the earliest mutations [7]. In patients with MPN or AML, *TET2* was found to co-mutate with *JAK2V617F*, *ASXL1*, *SRSF2*, *SF3B1*, *NPM1*, *FLT3* and *DNMT3A* [7, 14].

Recent studies demonstrated that inflammatory signals accelerate leukemogenesis driven by *TET2* loss. In *Tet2*-deficient mice, bacterial invasion due to a dysfunctional small-intestinal barrier was found to promote pre-leukemic myeloproliferation by increasing interleukin-6 (IL-6) production [15]. *TET2* loss was shown to confer a proliferative advantage on HSPCs after exposure to TNFα and IFNγ as compared to their wildtype HSPCs [16]. In response to inflammatory stress induced by lipopolysaccharide (LPS), *Tet2*-deficient HSPCs exhibited an increased resistance to apoptosis as well as enhanced production of IL-6 and hyperactivation of the Shp2-Stat3 signaling pathway, compared to wildtype HSPCs [17]. Targeting SHP2 or STAT3 with inhibitors suppressed the survival advantage of *Tet2*-deficient HSPCs [17]. These data implicate inflammation in the development of CH induced by TET2 deficiency.

Inflammation is a complex biological response that is induced by different kinds of stimulus [18], further investigation is needed to determine the mechanisms by which factors trigger the activation of inflammatory pathways and how these pathways contribute to the development of *TET2* mutant CH. To identify key factors responsible for this pathogenic process, we performed transcriptome analysis of distinct bone marrow populations in the hematopoietic systems of *Tet2* conditional knockout (hereafter named *Tet2*^*−/−*^) mouse models. The cGAS-STING pathway was found to be involved in dysregulated hematopoiesis and CH caused by mutated TET2. Deleting STING efficiently inhibited the expansion of LSK cells and the skewed myeloid differentiation in *Tet2*^*−/−*^ mice. We propose a novel mechanism involving the activation of the cGAS-STING pathway through which TET2 loss induces chronic inflammation in the hematopoietic system and impairs the homeostasis of HSPCs. Our finding also suggests that targeting STING could be a promising strategy to delay or prevent malignant transformation in healthy adults with CH who carry TET2 mutations.

## Materials and methods

### Mouse models

*Tet2*^*f/f*^ mice were generated as previously described [19]. *Sting*^−/−^ mice were kindly provided by Prof. Zhigang Lu [20]. *Mx1-Cre* (strain # 003556) and *Vav-Cre* (Strain # 008610) mice were purchased from Jackson Laboratory. *Tet2*^*f/f*^, *Mx1-Cre* and *Sting*^*−/−*^ mice were crossed to produce *Tet2*^*f/f*^, *Mx1-Cre* and *Tet2*^*f/f*^, *Sting*^*−/−*^, *Mx1-Cre* mice. To induce *Tet2* conditional knock-out, the Mx1-Cre transgene was induced in 4-week-old mice using five doses of poly(I:C) at 250 μg/body administered i.p. every other day. All mice were bred on a C57BL/6 genetic background. CD45.1 recipient mice (B6.SJL) were kindly provided by Prof. Xiaolong Liu. Immuno-deficient (B-NDG) mice were obtained from Biocytogen (Beijing, Cat. No. 110586) and used for establishing AML PDX models. All animal experiments described in this study were carried out according to the ethical guidelines of the Institute of Biochemistry and Cell Biology, Chinese Academy of Sciences, China.

### Bone marrow transplantation assays

Total BM cells were isolated respectively from *Tet2*^*f/f*^; *Sting*^*−/−*^; *Tet2*^*f/f*^, *Mx1-Cre*; *Tet2*^*f/f*^, *Sting*^*−/−*^, *Mx1-Cre*; *Tet2*^*f/f*^, *Vav-Cre* donor mice (CD45.2^+^). For non-competitive BM transplantations, 1 × 10^6^ CD45.2^+^ donor BM cells were transplanted into lethally irradiated (9.5 Gy) 8–10 weeks old CD45.1^+^ recipient mice through tail vein injection. For serial transplantations, 1 × 10^6^ BM cells were isolated from the previous recipients 18 weeks post-operatively and transplanted into lethally irradiated CD45.1^+^ recipients. For competitive transplantations, BM cells from different genotypes of donor mice (CD45.2^+^) and competitor mice (CD45.1^+^; B6.SJL) were mixed at a 1:1 ratio (0.5 × 10^6^ cells each) and injected into the tail vein of lethally irradiated (9.5 Gy) CD45.1^+^ recipient mice. Tail vein blood was collected from the recipient mice every four weeks after transplantation, and the nucleated blood cells were stained with antibodies against CD45.2, CD45.1, Mac1, Gr-1, CD4, and CD8 for FACS analysis.

### Reagents and antibodies

STING inhibitor C-176, C-178, and H-151 were purchased from MCE (Cat. No. HY-112906; HY-123963; HY-112693). 2′3′-cGAMP (Cat. No. SML1299) and ATP-^13^C_10_,^15^N_5_ (Cat. No. 645702) were obtained from Sigma. Due to the structural differences between mouse and human STING, C-178 and C-176 were used in mouse-based in vitro and in vivo analyses, while H-151 was exclusively utilized in experiments involving human samples. All other chemical reagents were purchased from Sigma or Sangon Biotech. Flag antibody (F3165), Tubulin antibody (SAB4500088), goat anti-rabbit (AP132), and goat-anti mouse secondary antibodies (AP124) were purchased from Sigma; all FACS antibodies were purchased from Invitrogen or BD; antibodies against MAVS (83000), STING (13647), p-STING (62912), and p-TBK1 (5483) were obtained from CST.

### Additional methods

Additional methods including lentivirus preparation, colony formation assay, limiting dilution assay, transduction of stem cells, flow cytometric analysis, immunofluorescence assay, PCR and real-time qPCR analysis, RNA library preparation and analysis, extraction and quantification of cGAMP, comet assay and statistical methods are provided in Supplementary methods.

## Results

### Activation of STING pathway in *Tet2-*deficient HSPCs

**Fig. 1:**
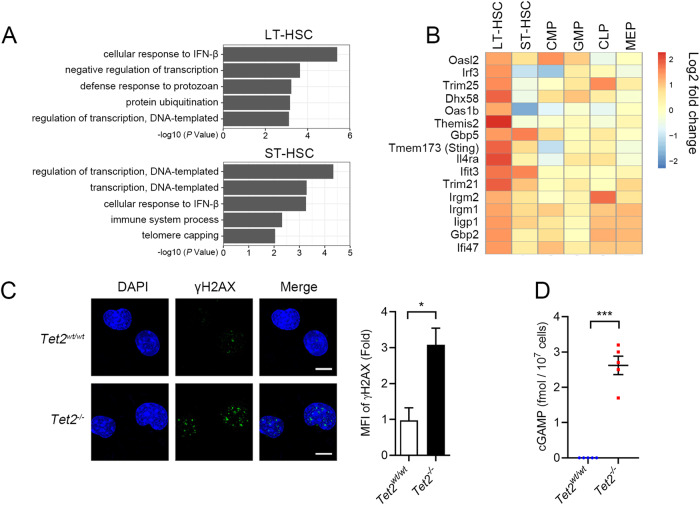
cGAS-STING pathway is activated in *Tet2*^*−/−*^ mouse HSPCs. **A** Gene sets enrichment analysis of differentially expressed genes showing upregulation of type I interferon response in *Tet2*^*−/−*^ hematopoietic stem cells (HSCs) compared to WT HSCs. There were 699 and 616 upregulated genes in *Tet2*-deficient LT-HSCs and ST-HSCs, respectively, compared to WT cells. DEGs were submitted to DAVID 6.7 (https://david.ncifcrf.gov) for gene ontology (GO) enrichment analyses (*n* = 4 mice, *p* < 0.05). **B** Heatmap showing activation of interferon- and inflammation-related genes in *Tet2*^*−/−*^ LT-HSCs (*P* < 0.05). Scale bar denotes log2-transformed fold change. Four replicates were analyzed for each cell type. **C** Immunofluorescence of *Tet2*^*−/−*^ LSK cells stained with γH2AX antibody (green). The quantification of γH2AX signal is shown to the right (MFI: Mean fluorescence intensity; scale bar, 5 μm). **D** cGAMP level in *Tet2*^*−/−*^ BM-MNCs quantified by LC-MS (*n* = 5 mice). Statistical significance was assessed by *t* test (**C**, **D**). Data are mean ± s.e.m., *P < 0.05; ***P < 0.005.

We performed RNA-seq of hematopoietic stem cells and lineage-restricted progenitor cells in bone marrow mononuclear cells (BM-MNCs) of wildtype (WT) and conditional knockout mice (Supplementary Fig. 1A, B). To avoid the interference from acute inflammatory stress potentially caused by poly(I:C) administration that was used for the induction of *Mx1*-Cre mediated gene knockout, mice were maintained for 16 weeks post induction before being sacrificed for analysis. The pathway enrichment analysis of differentially expressed genes (DEGs) revealed the involvement of type I interferon response specifically enriched in *Tet2*^*−/−*^ long term hematopoietic stem cells (LT-HSCs), short term hematopoietic stem cells (Fig. 1A) and megakaryocyte-erythroid progenitor cells (MEP) (Supplementary Fig. 1C), suggesting that the innate immunity-related pathways are activated in these cells. Among the upregulated innate immunity-related genes, STING [21–23], the key adapter of the cytosolic DNA sensing pathway (cGAS-STING pathway) [24], showed an expression change greater than 2-fold in *Tet2*^*−/−*^ HSCs compared to WT cells, especially in LT-HSCs (Fig. 1B and Supplementary Fig. 1D). Furthermore, the LT-HSCs displayed downregulated pathways upon the loss of *Tet2*, which were predominantly associated with metabolic processes, such as amide, organic cyclic compound, and nitrogen catabolic metabolic pathways (Supplementary Fig. 1E). After analyzing the altered pathways between WT and *Tet2*-deficient LT-HSCs, we hypothesized that the upregulation of innate immunity-related genes in *Tet2*^*−/−*^ HSCs could be attributed to the activation of the cGAS-STING pathway. This pathway is known to play a crucial role in sensing double-stranded DNA (dsDNA) and promoting the expression of inflammatory genes [25].

While infections are usually absent in the bone marrow (BM) environment of specific-pathogen-free mice, cellular DNA arising from genome instability represents a class of damage-associated molecular patterns which could activate the cGAS-STING pathway and stimulate the type I interferon response [26–28]. Indeed, double-strand breaks (DSBs) were increased in *Tet2*^*−/−*^ LSK cells compared with WT cells, as shown by anti-γH2AX immunofluorescence (Fig. 1C) and comet assay (Supplementary Fig. 1F). This is consistent with the disturbed expression of DNA damage response- and repair-related genes in *Tet2*^*−/−*^ LT-HSCs (Supplementary Fig. 1G). For instance, RNF138, along with other enzymes involved in the DNA repair-associated ubiquitin system, like UBE2a and UBE2D3, showed a significant reduction in *Tet2*-deficient LT-HSCs. The protein levels of STING and phosphorylated STING [29] and TBK1 [30], two biomarkers for activation of the STING-mediated type I interferon response, were increased in *Tet2*^*−/−*^ BM-MNCs (Supplementary Fig. 2A, B). Moreover, the expression of *Ifnβ* and *Il-6* was upregulated in *Tet2*^*−/−*^ LSK cells (Supplementary Fig. 2C). To determine whether the innate immune response in *Tet2*^*−/−*^ HSPCs is dependent on STING, we knocked down *Sting* and *Mavs* [31–34], crucial components of their respective cytosolic dsDNA/dsRNA sensing pathways, in *Tet2*^*−/−*^ LSK cells with shRNA. Notably, only *Sting* ablation dampened the upregulation of *Ifnβ* induced by *Tet2* deficiency (Supplementary Fig. 2D). It is known that cGAS triggers innate immune response through synthesis of cyclic GMP-AMP (cGAMP), which binds to and activates the adapter protein STING [35]. We then quantified the absolute amounts of cGAMP in the BM-MNCs from WT and *Tet2*^*−/−*^ mice using liquid chromatography-mass spectrometry (LC-MS). The amount of cGAMP in *Tet2*^*−/−*^ BM-MNCs reached 3 fmol/10^7^ cells, whereas it was undetectable in WT BM-MNCs (Fig. 1D and Supplementary Fig. 2E), confirming the activation of cGAS-STING pathway. Together, these data demonstrate that DNA damage induced by *Tet2* deficiency activates the cGAS-STING pathway, stimulating the downstream innate immune response in HSPCs.

### STING mediates skewed myelopoiesis and enhanced self-renewal of HSPCs with *Tet2* deletion

Previous studies reported that *Tet2*-deficient HSCs have a growth advantage, which can be increased by inflammatory cytokines, such as IL-6 [15] or TNF-α [36]. However, few studies to date have characterized the endogenous drivers of inflammation that contribute to the clonal expansion of *Tet2*-deficient HSPCs in the absence of an external inflammatory challenge such as microbial infection or LPS treatment. As cell-autonomous activation of the cGAS-STING pathway has been correlated with the expression of type I interferon and pro-inflammatory cytokines in *Tet2*^*−/−*^ HSPCs, it is tempting to speculate that blocking STING activation would suppress the increased self-renewal of these cells. We tested this hypothesis with the STING inhibitor C-178 [37] in a serial colony-forming unit (CFU) assay. Indeed, C-178 significantly reduced the replating capacity of *Tet2*^*−/−*^ LSK cells, and this effect was abrogated by IFNβ treatment (Supplementary Fig. 3A). To further eliminate the potential long-term inflammatory effects induced by poly(I:C) administration in the Mx1-Cre mediated *Tet2* conditional knockout system, we generated *Tet2*^*f/f*^, *Vav-Cre* (hereafter named *Tet2*^*−/−,Vav*^) mice to validate our results. Consistently, we found that C-178 treatment reduced the replating capacity of LSK cells obtained from *Tet2*^*−/−,Vav*^ mice (Supplementary Fig. 3B). The inhibitory effect of C-178 appeared specific to the LSK cells from *Tet2*^*−/−*^ mice as LSK cells from MLL-AF9 [38] or Nup98-Hox13 (NHD13) [39] transgenic mice could not be inhibited (Supplementary Fig. 3C). The C-178 inhibitory effect was correlated with cGAS-STING activation in response to DNA damage, as increased γH2AX signal and elevated expression of *Ifnβ* and *Il-6* were observed in cKit^+^ *Tet2*^*−/−*^ cells but not in WT, MLL-AF9- or NHD13-harboring cells (Supplementary Fig. 3D, E).

**Fig. 2:**
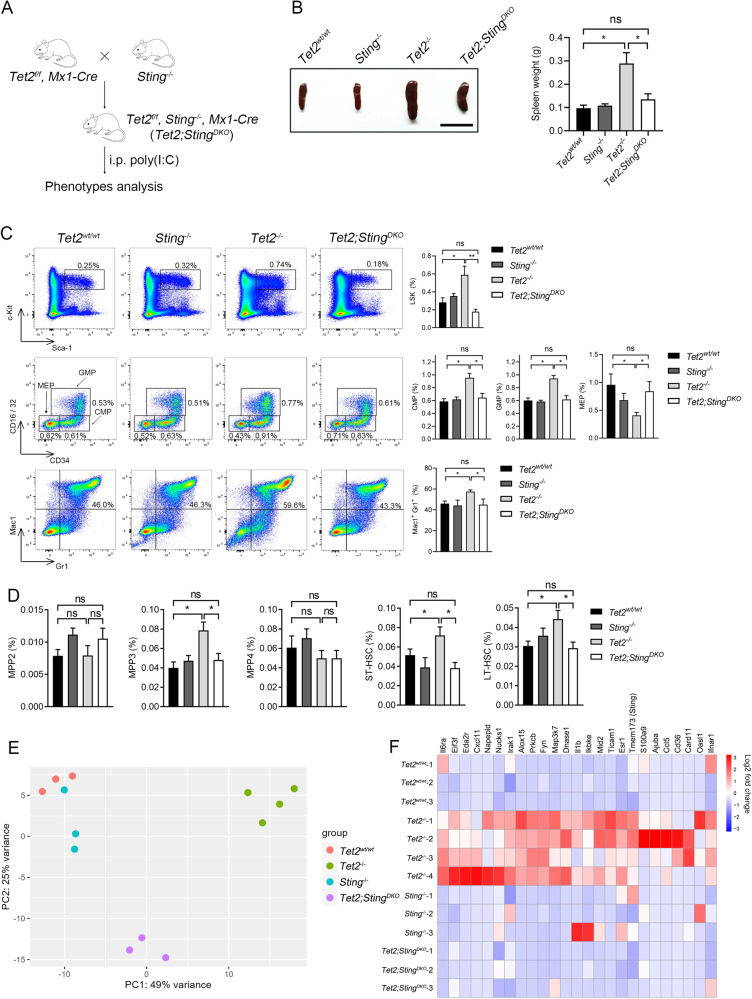
*Sting* is required for the increased self-renewal and myeloid-skewed differentiation of *Tet2*^*−/−*^ mouse HSPCs. **A** Breeding strategy for inducible deletion of *Tet2* in the hematopoietic system on the *Sting*^−/−^ background. i.p., intraperitoneal injection. **B** Comparison of spleens from representative mice of indicated genotypes. Shown on the right is the quantification of spleen weight (*n* = 5 mice; scale bar, 1 cm). **C** *Sting* deletion attenuates the aberrant hematopoiesis caused by *Tet2* deficiency. The percentages of LSK cells (top panel), oligopotent progenitor cells (CMP, GMP, and MEP, middle panel) and Mac1^+^Gr1^+^ myeloid cells (bottom panel) in the BM of indicated mice were determined by flow cytometric analysis. All mice were sacrificed for analysis 12 months after poly(I:C) injection. The quantification is shown to the right (*n* = 5 mice). **D** Deletion of *Sting* inhibits the expansion of MPP3 cell population and the enhanced self-renewal of HSCs in *Tet2*-deficient mice. The percentages of multipotent progenitor cells (MPP2/3/4), ST-HSCs, and LT-HSCs in the BM of indicated mice were measured by FACS (n = 8 mice). **E** PCA (Principal Component Analysis) plot showing the RNA-seq data of LT-HSCs derived from indicated genotypes of mice. Each dot represents an individual mouse (For *Tet2*^*−/−*^, *n* = 4; for other genotypes, *n* = 3). (**F**) Deletion of *Sting* attenuates the upregulation of innate immunity- and inflammation-related genes in LT-HSCs induced by *Tet2* deficiency. Genes with log2(fold change) >1 and *P* < 0.05 are shown (For *Tet2*^*−/−*^, *n* = 4; for other genotypes, *n* = 3). LT-HSC, long term hematopoietic stem cell; ST-HSC, short term HSC; CMP, common myeloid progenitor; GMP, granulocyte macrophage progenitor; CLP, common lymphoid progenitor; MEP, megakaryocyte erythroid progenitor. Statistical significance was assessed by *t* test (**B**–**D**). Data are mean ± s.e.m., *P < 0.05; **P < 0.01; “ns”: not significant.

Because the serial replating capacity of *Tet2*^*−/−*^ HSPCs was significantly reduced by C-178, we reasoned that blocking the cGAS-STING pathway may alleviate *Tet2* deficiency-induced expansion of HSPCs and aberrant myelopoiesis. To examine this possibility, we crossed *Tet2*^*f/f*^*, Mx1-Cre* with *Sting*^*−/−*^ to generate *Tet2*^*f/f*^, *Sting*^*−/−*^, *Mx1-Cre* mice. By administering poly(I:C) to these mice every other day for 10 days, we obtained double knockout mice for *Tet2* and *Sting* (hereafter named *Tet2*;*Sting*^*DKO*^) (Fig. 2A). Loss of STING was confirmed in the *Sting*^*−/−*^ and *Tet2*;*Sting*^*DKO*^ BM-MNCs (Supplementary Fig. 4A). *Ifnβ* and *Il-6* were significantly downregulated in the primary cKit^+^ cells isolated from *Tet2*;*Sting*^*DKO*^ BM compared with *Tet2*^*−/−*^ cells, indicating that the cGAS-STING pathway is required for the induction of these cytokines (Supplementary Fig. 4B). Moreover, the growth advantage of *Tet2*^*−/−*^ cKit^+^ cells was reduced after *Sting* deletion (Supplementary Fig. 4C). The replating capacity of *Tet2*;*Sting*^*DKO*^ LSK cells was also significantly reduced compared with that of *Tet2*^*−/−*^ cells, and IFNβ treatment could restore the replating capacity of *Tet2*;*Sting*^*DKO*^ LSK cells (Supplementary Fig. 4D). Collectively, these data suggest that an activated cGAS-STING pathway functions to establish a local inflammatory environment which supports and stimulates the self-renewal of *Tet2*^*−/−*^ HSPCs.

Previous studies have shown that inactivation of *Tet2* leads to an increased LSK pool and differentiation skewing towards monocytic/granulocytic lineages [10–12]. We then asked whether these phenotypes can be alleviated by additional *Sting* deletion. At 12 months post poly(I:C) induction, the size and weight of spleens in *Tet2*;*Sting*^*DKO*^ mice were significantly reduced compared with those from *Tet2*^*−/−*^ mice (Fig. 2B). We further examined myeloid differentiation and erythropoiesis in the peripheral blood (PB). Notably, myeloid-skewing induced by *Tet2* deficiency was attenuated by *Sting* deletion (Supplementary Fig. 4E), and reduced erythropoiesis, another typical phenotype of *Tet2*^*−/−*^ mice [40], was fully restored by *Sting* deletion (Supplementary Fig. 4F). *Sting* loss appeared to affect specifically the *Tet2*^*−/−*^ phenotypes because *Sting*-KO alone had no discernible effect on lineage differentiation.

We observed significantly more LSK cells in the BM of *Tet2*^*−/−*^ mice. However, there was almost no change in the LSK population when both *Tet2* and *Sting* were deleted. Furthermore, targeted deletion of *Sting* rescued the alteration of oligopotent progenitor cells in *Tet2*^*−/−*^ mice, including the increase of CMP and GMP and the decrease of MEP (Fig. 2C). Moreover, *Tet2*;*Sting*^*DKO*^ mice exhibited a comparable proportion of myeloid cells to *Tet2*^*wt/wt*^ control mice, and the expansion of myeloid cells in the BM was only observed in *Tet2*^*−/−*^ mice (Fig. 2C). Thus, cGAS-STING activation is required for the *Tet2* deficiency-induced expansion of myeloid progenitor cells and *Sting* deletion reduces the skewed differentiation of oligopotent progenitor cells. We also analyzed the populations of different MPP (multipotent progenitor cell) and HSCs in each genotype. MPP3 represents a distinct myeloid-skewed subpopulation, while MPP2 preferentially generates megakaryocyte/erythroid lineages and MPP4 produces lymphoid as well as myeloid lineages under regenerative conditions of the hematopoietic system [41]. We observed a significant increase of MPP3 cells in the bone marrow of *Tet2*^*−/−*^ mice, while MPP2/4 showed a similar proportion to *Tet2*^*wt/wt*^ mice. The unbalanced production of MPP cells was corrected in the bone marrow of *Tet2*;*Sting*^*DKO*^ mice, suggesting that the expansion of MPP3 cells in *Tet2*^*−/−*^ mice is dependent on *Sting*. The HSC pool was enlarged as a result of *Tet2* deletion, as evidenced by the significantly increased proportion of both ST- and LT- HSCs in the bone marrow of *Tet2*^*−/−*^ mice compared to WT mice. Similar to the effects observed in MPP3 cells, deletion of *Sting* reduced the expansion of the HSC pool in *Tet2*^*−/−*^ mice (Fig. 2D and Supplementary Fig. 4G) Together, these data suggest that the defects in hematopoiesis and myeloid skewing induced by *Tet2* deficiency are mediated by STING.

To investigate the molecular mechanism by which the cGAS-STING pathway is involved in *Tet2*-deficiency-mediated hematopoietic phenotypes, we performed RNA-seq on LT-HSCs derived from *Tet2*^*wt/wt*^, *Sting*^*−/−*^, *Tet2*^*−/−*^, and *Tet2*;*Sting*^*DKO*^ mice. Principal Component Analysis (PCA) showed that the mRNA expression profile in *Tet2*;*Sting*^*DKO*^ LT-HSCs was significantly different from that of *Tet2*^*−/−*^ mice (Fig. 2E). Deletion of *Sting* was found to significantly suppress the activation of inflammatory genes that were induced in *Tet2*^*−/−*^ LT-HSCs (Fig. 2F). We also conducted GO and KEGG analysis for DEGs between *Tet2*^*−/−*^ versus *Tet2*;*Sting*^*DKO*^ LT-HSCs (Supplementary Fig. 4H, I). Among the pathways analyzed, the small GTPase signal transduction pathway was found to be the most upregulated in *Tet2*^*−/−*^ LT-HSCs compared to DKO cells, whereas the RNA splicing process was identified as the most downregulated pathway in *Tet2*-deficient cells. These data indicate that the upregulation of inflammatory cytokines in *Tet2*-deficient LT-HSCs depends on the activation of the cGAS-STING pathway, and various signaling pathways are involved in the dysregulated hematopoiesis upon the loss of *Tet2*.

### STING promotes hematopoietic disorders induced by *Tet2* loss

**Fig. 3:**
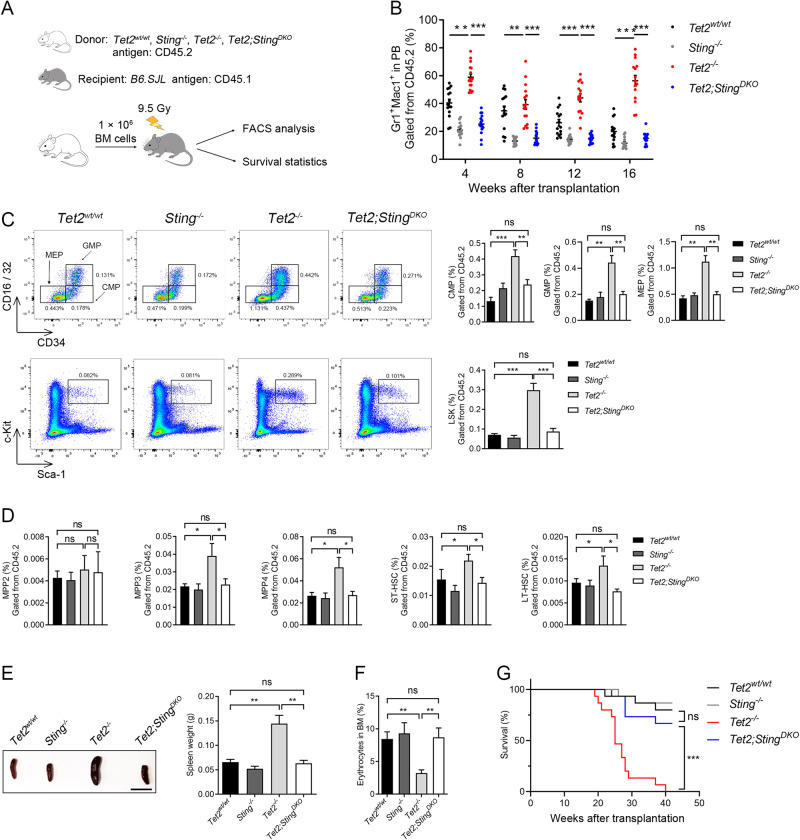
*Sting* deletion abrogates *Tet2* deficiency-induced skewed hematopoiesis in bone marrow transplantation models. **A** Schematic of bone marrow transplantation assay. **B** Myeloid skewing of *Tet2*^*−/−*^ cells after transplant (red dots) is corrected by *Sting* (blue dots) deletion. The scatter plot shows the quantification of donor-derived mature myeloid cells in the peripheral blood (PB) of primary recipients transplanted with indicated donor BM cells. **C** *Sting* deletion attenuates the expansion of *Tet2*^*−/−*^ progenitor cells in recipient mice. Representative FACS plots of progenitor cells from the BM cells of indicated donor groups are shown on the left. Top row, CMP, GMP, MEP; Bottom row, LSK. Quantification is shown on the right. **D** Bar graphs showing the populations of MPP cells and HSCs in recipient mice analyzed by FACS. **E** Comparison of spleens from recipient mice of indicated genotypes. Shown to the right is the quantification of spleen weight. (*n* = 3 donors and 15 recipients for each genotype; scale bar, 1 cm). **F** *Sting* deletion restores normal erythropoiesis of *Tet2*^*−/−*^ donors. The proportion of Ter119^+^ erythrocytes in the BM were analyzed by FACS. **G** Survival analysis of primary transplant recipients of indicated donor groups (*n* = 3 donors and 15 recipients for each genotype). In BM transplantation assays, all mice (*n* = 3 donors and 15 recipients for each genotype) were sacrificed and analyzed at 18 weeks after transplantation. Statistical significance was assessed with *t*-test (**B**–**F**), and Mantel–Cox log-rank test (G). Data are mean ± s.e.m., *P < 0.05; **P < 0.01; ***P < 0.005; “ns”: not significant.

In a non-competitive transplantation assay (Fig. 3A, Supplementary Fig. 4J), *Tet2*^*−/−*^ BM donor cells exhibited skewed differentiation towards the myeloid lineage in the PB of recipient mice, whereas the myeloid skewing phenotype was ameliorated after *Sting* deletion (Fig. 3B). Eighteen weeks after transplantation, recipients were sacrificed for analysis. Consistent with significant myeloid expansion, the populations of oligopotent progenitors (CMP, GMP, and MEP) and LSK cells from *Tet2*^*−/−*^ donors had increased more than 2-fold relative to those from *Tet2*^*wt/wt*^ control donors. In contrast, these cell proportions were not significantly changed in mice who had received cells *Tet2*;*Sting*^*DKO*^ donors (Fig. 3C). In the recipients of *Tet2*^−/−^ donors, the proportion of MPP3/4 was 2-fold higher than that in *Tet2*^*wt/wt*^ or *Tet2*;*Sting*^*DKO*^ donors, and the population of MPP2 showed no difference among all donors (Fig. 3D). The proportion of HSCs (ST- or LT-HSC) was increased by 50% in recipients of *Tet2*^*−/−*^ donors. In contrast, recipients of *Tet2*;*Sting*^*DKO*^ donors exhibited normal-sized HSC populations. (Fig. 3D). We observed that the phenotype of enlarged spleen and compromised differentiation potential towards erythroid lineage seen in recipient of cells from *Tet2*^*−/−*^ donors was rescued in recipients of cells from *Tet2*;*Sting*^*DKO*^ donors (Fig. 3E, F), suggesting that the *Tet2;Sting*^*DKO*^ donor cells sustained grossly normal hematopoiesis. Strikingly, *Sting* deletion significantly prolonged the survival of recipients of *Tet2*^*−/−*^ BM cells (Fig. 3G), indicating that targeting STING might represent a novel therapeutic approach to inhibit the onset of leukemogenesis driven by mutated *TET2*. Taken together, the above data suggest that additional deletion of *Sting* rescues both the function and homeostasis of the hematopoietic system of *Tet2*^*−/−*^ mice.

**Fig. 4:**
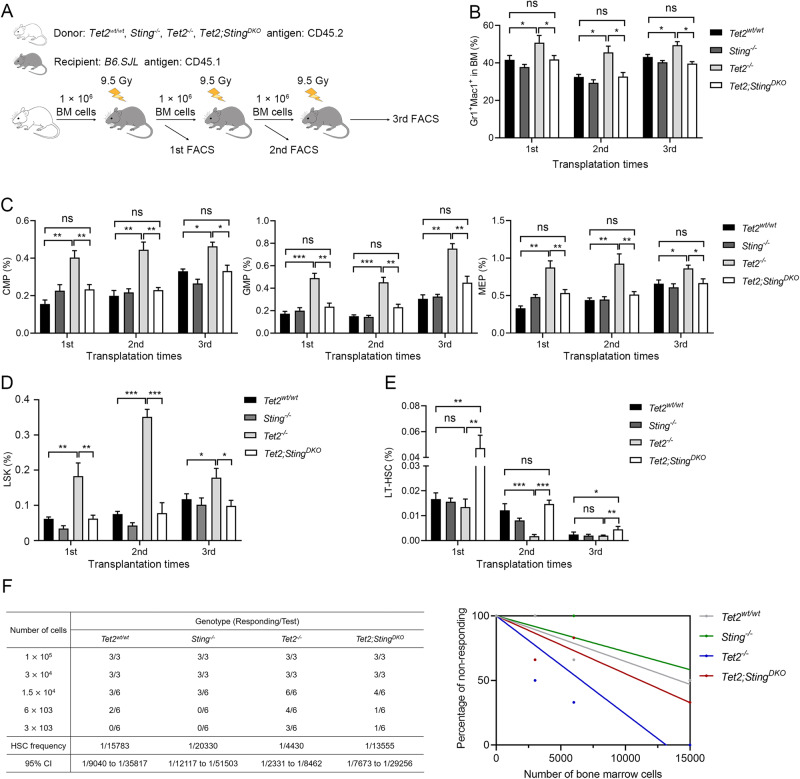
Blocking STING restores normal hematopoiesis of *Tet2*^*−/−*^ BM cells in serial transplantation models. **A** Schematic of serial transplantation assays. **B** Myeloid skewing of *Tet2*^*−/−*^ BM donors is corrected by *Sting* deletion. **C** *Sting* deletion attenuates the expansion of transplanted *Tet2*^*−/−*^ progenitor cells in recipient mice. **D** *Sting* deletion mitigates the abnormal expansion of *Tet2*^*−/−*^ LSK cells in serial transplantation recipients. **E** *Sting* deletion increases the proportion of *Tet2*^*−/−*^ donor LT-HSCs in serial transplantation recipients. **F** The frequencies of functional HSCs in each genotype of mice were measured by the limiting dilution assay. Table to the left displays the cell dose, responding rate, calculated HSC frequencies and 95% confidence interval (CI), while the Poisson statistical analysis is presented on the left, color lines indicate the best best-fit linear model. The frequencies and analysis were performed using the L-Calc software. All mice (*n* = 2 donors and 10 recipients for each genotype) in the serial transplantation assay were sacrificed and analyzed 18 weeks after transplantation. Statistical significance was assessed with two-way ANOVA (**B**–**D**) and *t* test (**E**). Data are mean ± s.e.m., *P < 0.05; **P < 0.01; ***P < 0.005; “ns”: not significant.

To further investigate whether STING is required for the increased self-renewal of *Tet2*^*−/−*^ HSPCs, we performed three rounds of serial transplantation, each lasting for 16 weeks (Fig. 4A, Supplementary Fig. 4H). The increase in the population of *Tet2*^*−/−*^ progenitor cells derived from donor mice was abrogated by *Sting* deletion (Fig. 4B and C). Additionally, while *Tet2*^*−/−*^ donors generated 0.5-3.5-fold more LSK cells than *Tet2*^*wt/wt*^ donors in each round of transplantation, LSK cells were not increased in the recipients of cells from *Tet2*;*Sting*^*DKO*^ donors (Fig. 4D). Interestingly, although the frequency of LT-HSCs usually decreased after each round of transplantation, the LT-HSC population in the recipients of *Tet2*;*Sting*^*DKO*^ donors was larger than that of WT and *Tet2*^*−/−*^ donors (Fig. 4E). In has been shown that *Tet2*-deficient HSCs exhibited an enhanced potential for regenerating the hematopoietic system during serial transplantation assays [42]. It would be interesting to examine whether blocking the STING pathway in *Tet2*-deficient hematopoietic cells can further increase the functional HSC pool in the bone marrow. To investigate this question, we used a limiting dilution assay to measure the frequencies of HSCs in different donor genotypes. Surprisingly, even though *Tet2*;*Sting*^*DKO*^ donors had two-fold more HSCs than *Tet2*^*−/−*^ donors in the serial transplantation assay, the frequency of functional HSCs in their bone marrow was lower than that in *Tet2*^*−/−*^ donors: 1 HSC in 13,555 bone marrow cells compared to 1 in 4430 (Fig. 4F). This result further supports the notion that deleting the *Sting* impairs the reconstitution capacity of *Tet2*^*−/−*^ HSCs, and suggests that blocking STING might affect the stem cell pool in TET2-mutation-associated hematological disorders.

To investigate whether STING is essential for the enhanced self-renewal activity of *Tet2*^*−/−*^ HSPCs, we performed competitive transplantation assays (Supplementary Fig. 5A, Supplementary Fig. 4H). As expected, *Tet2*;*Sting*^*DKO*^ BM donor cells showed a significantly reduced level of reconstitution capacity compared to *Tet2*^*−/−*^ cells (Supplementary Fig. 5B), indicating that *Sting* contributes to the competitive advantage conferred by *Tet2* deficiency. In the PB, the skewed myelopoiesis observed in *Tet2*^*−/−*^ mice was corrected by deletion of *Sting* (Supplementary Fig. 5C). *Tet2*;*Sting*^*DKO*^ mice showed comparable populations of myeloid cells, LSK cells, and LT-HSCs in the bone marrow with *Tet2*^*wt/wt*^ donors. Meanwhile, *Tet2*-deficient donors exhibited increased myeloid cells and expanded HSPC pools compared to other genotypes of donors in the recipient mice (Supplementary Fig. 5D). To further test whether STING inhibition can suppress the competitive advantage of donor cells derived from *Tet2*^*−/−,Vav*^ mice, we co-transplanted *Tet2*^*−/−,Vav*^ and CD45.1 BM cells into recipient mice and treated them with the STING inhibitor C-176, a water soluble small molecule suitable for in vivo assays [37]. Similar to genetic deletion models, pharmacological inhibition of STING reduced the proportion of *Tet2*^*−/−,Vav*^ donor cells in the PB of recipient mice by approximately 15%, compared to the DMSO group. (Supplementary Fig. 5E). When we isolated the BM cells of recipient mice for analysis, we observed a decrease in LSK and LT-HSC populations in C-176-treated *Tet2*^*−/−,Vav*^ mice comparing to those treated with DMSO, with no influence on WT donor cells (Supplementary Fig. 5F). Treatment with C-176 also alleviated the skewed hematopoietic differentiation of *Tet2*-deficient donors, as evidenced by an increase in erythrocytes and a decrease in myeloid cells in *Tet2*^*−/−,Vav*^ donors compared to those treated with DMSO (Supplementary Fig. 5G).

Collectively, these data demonstrate that *Sting* is a key factor underlying *Tet2*-loss-induced hematopoietic disorders, and that blocking the cGAS-STING pathway by either gene deletion or small molecules can restrain increased self-renewal and skewed differentiation of HSPCs induced by *Tet2* deficiency.

### Blocking STING suppresses AML harboring *TET2* mutation

**Fig. 5:**
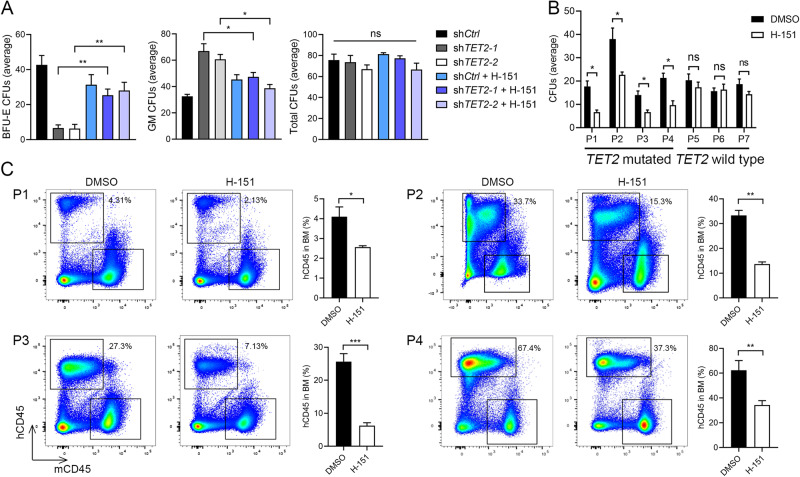
Myeloid skewing of TET2-deficient human HSCs and leukemogenesis of TET2-mutated AML Patient cells are prevented by inhibiting STING. **A** STING inhibitor H-151 reduces myeloid colony formation and restores erythroid colony formation of TET2-deficient cord blood CD34^+^ cells. CD34^+^ cells were infected with lentivirus carrying short hairpin RNA which targeted *TET2* (sh*TET2-1* or sh*TET2-2*) or scrambled control (sh*Ctrl*). Cells were cultured in methylcellulose medium for 14 days. **B** H-151 inhibits colony formation of *TET2*-mutated patient AML cells. P1-4 are AML patients with *TET2* mutations, and P5-7 are those without *TET2* mutation. **C** H-151 reduces the engraftment efficiency of *TET2*-mutated patient AML cells in B-NDG mice. The percentages of human CD45-positive cells (hCD45) in total BM are indicated. Statistical significance was assessed by *t* test (**A**–**C**). Data are mean ± s.e.m., *P < 0.05; **P < 0.01; ***P < 0.005; “ns”: not significant.

CHs harboring *Tet2* mutation alone rarely progress to hematopoietic malignancies [43]; cooperating mutations [44], such as in *Flt3* or *Jak2*^*V617F*^, accelerate leukemogenesis in mouse models [45, 46]. As the recipient mice of *Tet2*;*Sting*^*DKO*^ BM donor cells exhibited normal hematopoietic hierarchy and significantly extended survival, we wondered whether STING inhibition is able to restrain the skewed differentiation and leukemia progression of *TET2*-mutated human blood cells in vitro and in engrafted mice. We first examined the effect of human STING inhibitor H-151 [37] on the differentiation of human cord blood-derived CD34^+^ HSPCs upon *TET2* depletion. Consistent with a previous study [47], ablating TET2 with shRNA increased the granulocyte-macrophage colony-forming unit (CFU-GM) but reduced the burst forming unit-erythroid compared with the control group. The CD34^+^ cells with TET2 ablation showed upregulated IFNβ and increased micronuclei release (Supplementary Fig. 6A, B). Furthermore, the cGAS protein exactly co-localized with the micronuclei in the cytoplasm, suggesting that DNA damage induced by TET2 ablation activates the cGAS-STING pathway (Supplementary Fig. 6C). Notably, H-151 treatment attenuated the CFU-GM colony formation and restored erythroid colony formation of TET2-ablated cells (Fig. 5A). Consistently, knockdown of STING normalized IFNβ expression and lineage-skewed colony formation in TET2-ablated CD34^+^ cells (Supplementary Fig. 7A, B).

To evaluate the therapeutic potential of STING inhibition, we tested the effect of H-151 on colony formation of BM cells derived from 4 AML patients with *TET2* mutations (P1-4) and 3 AML patients without *TET2* mutations (P5-7) (Supplementary Fig. 8A). RT-qPCR analysis showed that while *TET2* mRNA levels were reduced, the expression levels of inflammatory genes were increased in P1-4 cells compared with P5-7, and the in vitro colony formation of P1-4 was inhibited by H-151 whereas that of P5-7 was not affected (Fig. 5B and Supplementary Fig. 8B). We further established PDX models in immunodeficient mice using mononuclear cells from these patients. The engraftment was examined 4 weeks after transplantation, and mice were treated with H-151 from 12 weeks onwards. In the PDX models of P5-7 without *TET2* mutations, the patient-derived CD45^+^ cells continued to expand in the PB during H-151 treatment; in contrast, the proportions of P1-4-derived hCD45^+^ cells in the PB were significantly reduced by H-151 administration (Supplementary Fig. 8C), indicating that H-151 specifically inhibits the leukemogenesis of *TET2*-mutated patient cells. Furthermore, compared to the DMSO group, the proliferation of *TET2*-mutated leukemia cells was significantly reduced in the BM of recipient mice treated with H-151, and no inhibitory effect was observed in the recipients of leukemia cells harboring wildtype *TET2* (Fig. 5C and Supplementary Fig. 8D). These data indicate that the leukemogenic potential of *TET2*-mutated AML mononuclear cells can be suppressed by inhibiting STING.

## Discussion

**Fig. 6:**
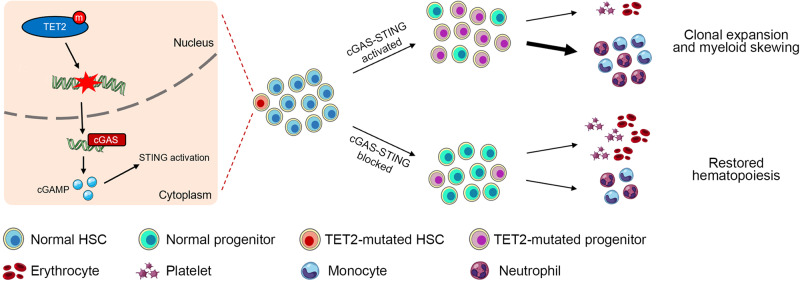
Working model for the activation of STING in mediating TET2 deficiency-associated CH. Cartoon summarizing our findings: HSPCs with mutated *TET2* accumulate DNA damage, which in turn activates the cGAS-STING pathway leading to the production of inflammatory cytokines. This promotes clonal expansion and myeloid differentiation skewing of the mutant HSPCs. Blocking the cGAS-STING pathway suppresses the increased self-renewal and skewed differentiation potential of the mutant HSPCs, and thus restores normal hematopoiesis.

In this study, we demonstrated that DNA damage engendered by TET2 deficiency activated the cGAS-STING pathway in HSPCs. The specific mechanism underlying DNA damage induction in *Tet2*^*−/−*^ HSPCs is not yet clear. However, we observed altered expression of several DNA repair-associated genes in *Tet2*^*−/−*^ HSPCs compared to WT cells, indicating that the genome becomes unstable due to insufficient repair upon loss of *Tet2*. Moreover, inhibition of STING significantly attenuated the expansion of LSK and skewed myeloid differentiation in both *Tet2*^−/−^ mice and bone marrow transplant models. Compared to *Tet2*^−/−^ donor cells, *Tet2*;*Sting*^*DKO*^ donor cells restored a balanced hematopoietic hierarchy and extended significantly the lifespan of recipient mice. Leveraging AML patient-derived xenograft models, we further showed that patient-derived BM-MNCs with *TET2* mutations were more sensitive than those without *TET2* mutations to STING inhibition. Taken together, these data suggest that STING plays an essential role in hematopoietic disorders induced by *TET2* mutation and inhibition of STING represents a potential therapeutic strategy to block the production of inflammatory cytokines and mitigate the development of CH driven by *TET2* mutations (Fig. 6).

Chronic low-grade inflammation promotes myeloid differentiation and leukemogenesis in *Tet2*-deficient mice [17, 36]. Using microbial infection- or LPS injection-induced inflammatory models, several studies have shown that the hematopoietic malignancies driven by *TET2* mutation were effectively prevented by targeting the activated inflammatory pathways [15, 17, 48]. These observations showed that inflammation accelerates myeloid transformation on the basis of *TET2* deficiency. However, the mechanisms by which hematopoietic disorders develop spontaneously in *Tet2* knockout specific pathogen-free mice have remained unclear. Here we show that the damaged DNA from genome instability activates the cGAS-STING pathway and induces a sterile inflammatory reaction in *Tet2*^*−/−*^ HSPCs, which in turn fosters an inflammatory environment and promotes the dysregulated hematopoiesis. This indicates that the development of CH and leukemic transformation of TET2-mutated HSPCs can be promoted not only by an acute inflammatory response triggered by external stimuli, but also by chronic inflammation induced by internal stimuli in mutated HSPCs. Recently, a study showed that the activation of the cGAS/STING/NLRP3 axis by cytoplasmic DNA in *Tet2*-deficient hematopoietic stem/progenitor cell line promotes myelodysplastic neoplasms development [49]. Taking account their findings and our own, inhibiting STING at either early or pathological stage has the potential to effectively suppress the aberrant hematopoiesis induced by TET2 mutations. Similar to TET2, another DNA modification enzyme, DNA methyltransferase 3 Alpha (DNMT3A), which frequently mutates in hematopoietic malignancies, is also considered a driver mutation of CH. Recent work indicates that inflammatory signals promote the development of *Dnmt3a* mutation-associated CH [50], and the phenotypes are similar as those in *Tet2* mutant CH. In macrophages, loss of function of DNMT3A or TET2 both induces type I interferon and other inflammatory signals through mitochondria DNA-activated cGAS-STING pathway [51]. In our AML patient samples, two of them harbor DNMT3A mutations, but they did not show any response to H-151 treatment. However, one of the mutations is a synonymous mutation, and the other is not a common hot spot mutation observed in AML patients. We hypothesized that these mutations may not directly impact the catalytic activity of DNMT3A. It would be interesting to investigate whether the cGAS-STING pathway is activated in hematopoietic disorders associated with loss-of-function mutations in DNMT3A, such as the R882H mutation, as well as in other hematopoietic malignancies characterized by genome instabilities. Moreover, exploring the effect of targeting the cGAS-STING pathway to prevent the development of these diseases are of potential clinical relevance.

The cGAS-STING pathway has emerged as a crucial pathway in cancer immunology. Recent studies report that activation of the cGAS-STING pathway can be used to enhance antitumor immunotherapy [52, 53], in part due to the induction of type I interferons which enhance the recognition of tumor cells and the killing efficiency of T cells [54]. However, activation of the STING pathway also enhances the IL-6-dependent survival of chromosomally unstable breast cancer cells, suggesting a pro-tumorigenic effect of cGAS-STING signaling [55]. Therefore, the effect of an activated cGAS-STING pathway can be ambiguous. Previous studies have shown that activating STING can induce apoptosis in lymphocytes and monocytes [56–58]. These studies employed either cGAMP, 10-carboxymethyl-9-acridanone, or 5,6-dimethylxanthenone-4-acetic acid as agonizts to induce a potent activation of the cGAS/STING pathway in cells (more than 20-fold increase), resulting in acute and pronounced inflammatory responses both in vitro and in vivo. In our study, *Tet2* deficiency-induced DNA damage leads to a chronic and mild activation of the cGAS/STING pathway and inflammatory response (about 3 folds). Furthermore, it has been proposed that chronic inflammatory responses are associated with cellular mutations and increased proliferation [59]. These observations suggest that the extent and duration of the inflammatory response induced may play a pivotal role in determining the diverse outcomes of STING activation in cells.

In summary, our study uncovers an unexpected role of STING in CH mediated by TET2 deficiency and demonstrates that targeting STING can effectively restore the normal hematopoiesis in *TET2*-mutated mice. These findings provide new insights into the development of CH and an exciting new opportunity to develop therapeutic strategies to delay or stop this process.

### Supplementary information

Supplementary Figure legends and addtional methods

Supplementary Figure 1

Supplementary Figure 2

Supplementary Figure 3

Supplementary Figure 4

Supplementary Figure 5

Supplementary Figure 6

Supplementary Figure 7

Supplementary Figure 8

Dataset1

Dataset2

## Data Availability

The data supporting the findings of this study are provided within the article. Raw RNA sequencing data have been deposited in the GEO database under accession number GSE232384. Additional information related to this research is available upon request from the corresponding author.
