# Supplementary material for: STING activation in TET2-mutated hematopoietic stem/progenitor cells contributes to the increased self-renewal and neoplastic transformation
Source: Leukemia. 2023 Oct 10;37(12):2457–67. doi: 10.1038/s41375-023-02055-z (PMC10681905; doi:10.1038/s41375-023-02055-z)
Supplement: Supplementary file 1 — Supplementary Figure legends and addtional methods [file 41375_2023_2055_MOESM1_ESM.docx]

Manuscripts: Jiaying Xie et al.

**STING activation in TET2-mutated hematopoietic stem/progenitor cells contributes to the increased self-renewal and neoplastic transformation**

**Supplemental information:**

Supplemental figures and additional methods are provided in supplemental data.

**Supplemental figures**

**Supplementary figure 1. Activated innate immunity pathway and increased DNA damage in hematopoietic stem cells of *Tet2*^-/-^ mouse.**

(A) Schematic of Cre-mediated *Tet2* gene conditional knockout. Coding exons are shown as filled boxes. LoxP sites are shown as yellow triangles and genotyping primers as arrows. The positions of the catalytic active site of TET2 are marked by a star. Panel to the right shows the genotyping primers and the result of Cre-mediated *Tet2* deletion.

(B) Gating strategy used for sorting hematopoietic stem cells and oligopotent progenitor cells from bone marrows. LT-HSC, Lin^-^cKit^+^Sca1^+^CD48^-^CD150^+^; ST-HSC, Lin^-^cKit^+^Sca1^+^CD48^-^CD150^-^; CMP, Lin^-^cKit^+^Sca1^-^CD34^int/+^CD16/32^int^; GMP, Lin^-^cKit^+^Sca1^-^CD34^+^CD16/32^high^; MEP, Lin^-^cKit^+^Sca1^-^CD34^-^CD16/32^low^; CLP, Lin^-^cKit^+^Sca1^int^ CD127^+^

(C) Enrichment analysis of differentially expressed genes of upregulated pathways in *Tet2^-/-^* hematopoietic progenitor cells. There were 698, 545, 336 and 767 upregulated genes in *Tet2^-/-^* CMP, GMP, MEP and CLP cells, respectively, compared to WT cells. DEGs were submitted to DAVID 6.7 (https://david.ncifcrf.gov) for gene ontology (GO) enrichment analyses (*n* = 4 mice, *P* < 0.05) (see also Fig.1).

(D) Heatmap showing upregulated innate immunity- and inflammation-related genes in *Tet2^-/-^* LT-HSCs. The genes with log_2_(fold change) > 1 and *P* < 0.05 are shown (*n* = 4 mice).

(E) The pathways that were downregulated in the transcriptome analysis due to the loss of *Tet2* in LT-HSCs have been illustrated using plot diagrams The data from the GO analysis was shown on the left, while the KEGG analysis was shown on the right (n = 4 mice).

(F) Alkaline comet assay showing increased damaged DNA in *Tet2*^-/-^ LSK cells. Quantifications of cells with tails and tail moment are shown at bottom. (*n* = 3 mice, statistical significance was assessed by t-test, data are mean ± s.e.m., ****P* < 0.005)

(G) Heatmap showing the differential expression of DNA damage response-related genes in *Tet2^-/-^* LT-HSCs. Up- or down-regulation are defined as mean log_2_(fold change) > 1 and *P* < 0.05 (*n* = 4 mice).

**Supplementary figure 2. The STING-mediated innate immunity pathway is activated in *Tet2^-/-^* hematopoietic cells.­­**

(A) Western blot showing the upregulation of *Sting* in *Tet2^-/-^* BM-MNCs. MAVS and Tubulin were used for normalization.

(B) Phosphorylation of STING and TBK1 in *Tet2^-/-^* BM-MNCs was detected by Western blot. Tubulin was used for normalization.

(C) Upregulation of *Ifnβ* and *Il-6* in *Tet2^-/-^* LSK cells. The mRNA levels of *Ifnβ* and *Il-6* are quantified by qRT-PCR (n = 3 mice)

(D) *Sting* is required for type I interferon production in *Tet2^-/-^* LSK cells. The mRNA levels of *Sting*, *Mavs* and *Ifnβ* were quantified by qRT-PCR (n = 3 mice).

(E) Mass chromatograms displaying quantification of intracellular cGAMP (upper panel) in *Tet2*-deficient BM-MNCs. ^13^C_10_^15^N_5_-labeled cGAMP was spiked in the LC-MS samples as an internal standard (bottom panel) and the cGAMP peak is highlighted in yellow (n = 5 mice).

Statistical significance was assessed by t-test (C, D), data are mean ± s.e.m., ***P* < 0.01, ****P* < 0.005, “ns”: not significant.

**Supplementary figure 3. STING inhibitor C-178 inhibits the replating capacity of *Tet2*^-/-^ LSK cells specifically.**

(A) Reduction in the serial replating potential of *Tet2^-/-^* LSK cells cultured in the presence of C-178 (5μM). The addition of IFNβ treatment (0.2 U/ml) restored the replating ability of *Tet2^-/-^* LSK cells treated with C-178 (*n* = 3 mice).

(B) The serial repopulation capacity of LSK cells derived from *Tet2^-/-,Vav^* mice is inhibited by C-178 (n = 3 mice).

(C) C-178 treatment does not reduce the replating ability of MLL-AF9- or Nup98-Hox13 (NHD13)-transformed LSK cells (n = 3 independent experiments). Colony formation assay was performed with LSK cells derived from AML mouse models harboring the fusion oncoproteins of MLL-AF9 (left) or Nup98-Hox13 (right).

(D) Western blot showing the phosphorylation of H2AX in *Tet2^-/-^* cKit^+^ cells. H2AX and Tubulin were used as normalization

(E) Upregulation of *Ifnβ* and *Il-6* in *Tet2^-/-^* cKit^+^ cells. The mRNA levels of *Ifnβ* and *Il-6* were quantified by qRT-PCR (n = 3 mice).

Statistical significance was assessed by t-test (A-E). Data are mean ± s.e.m., ***P* < 0.01, ****P* < 0.005, “ns”: not significant.

**Supplementary figure 4. *Sting* deletion attenuates increased growth advantage and myeloid-skewed differentiation induced by *Tet2* deficiency.**

(A) Western analysis of STING expression in the BM cKit^+^ cells with indicated genotypes. Tubulin was used for normalization.

(B) *Sting* deletion mitigates the upregulation of *Ifnβ* and *Il-6* in *Tet2^-/-^* cKit^+^ cells. The mRNA levels of *Ifnβ* and *Il-6* were quantified by qRT-PCR (n = 3 mice).

(C) *Sting* deletion reduces the growth rate of *Tet2^-/-^* hematopoietic progenitor cells. cKit+ cells were isolated from BM cells of 16-week-old mice and cultured in SFEM medium supplemented with SCF (100 ng/ml), IL-3 (10 ng/ml) and IL-6 (10 ng/ml). Cell viability was measured by CCK8 assay (n = 3 mice, biological replicates).

(D) Reduction in the replating potential of isolated *Tet2^-/-^* LSK cells when lacking *Sting*. IFNβ treatment (0.2 U/ml) restored the replating ability of *Tet2;Sting^DKO^* LSK cells (*n* = 3 experiments, each group with 1 mouse/experiment).

(E) *Sting* deletion attenuates *Tet2-*deficiency induced myeloid-skewed differentiation in the peripheral blood (PB). CD4^+^ or CD8^+^ T cells represent differentiation of lymphocytes (n = 5 mice).

(F) *Sting* deletion restores normal erythropoiesis of *Tet2^-/-^* mice (n = 5 mice). The proportion of Ter119^+^ erythrocytes was analyzed by FACS.

(G) The antibody labeling and gating strategy used to identify MPP2/3/4, ST- and LT-HSC in the bone marrow cells.

(H) Upregulated pathways in *Tet2*-deficient LT-HSCs compared to DKO LT-HSCs. GO analysis is shown on the left, while KEGG analysis is shown on the right (n = 3)

(I) Downregulated pathways in *Tet2*-deficient LT-HSCs compared to DKO LT-HSCs. GO analysis is shown on the left, while KEGG analysis is shown on the right (n = 3)

(J) The genotypes of the donor mice used in non-competitive, serial, and competitive transplantation were confirmed by PCR analysis. 1 million CD45.2^+^ donor cells were sorted from total bone marrow of recipient mice by FACS, and the genomic DNA was extracted for genotyping.

All mice (E-F) were analyzed between 40 to 44 weeks of age. Statistical significance was assessed by t-test (B-F). Data are mean ± s.e.m., **P* < 0.05, ***P* < 0.01, ****P* < 0.005, “ns”: not significant.

**Supplementary figure 5. Targeting STING abrogates *Tet2* deficiency-induced clonal hematopoiesis in competitive BMT assays.**

(A) Schematic of cBMT assays.

(B) *Sting* (blue line) deletion reduces the competitive advantage of *Tet2^-/-^* donor cells (red line) in cBMT assay (2 donors and 10 recipients for each genotype).

(C) *Sting* deletion decreases the elevated population of myeloid cells in the peripheral blood of *Tet2*-deficient donors.

(D) Bar graphs showing the populations of myeloid cells, LSK cells, and LT-HSCs in the BM of cBMT recipient mice analyzed by FACS.

(E) STING inhibitor C-176 abrogates the competitive advantage of *Tet2^-/-,Vav^* donor cells in a cBMT assay (1 donor and 8 recipients). Mice were treated with C-176 or DMSO by i.p. every 3 days and engraftment of donor cells (CD45.2) was analyzed every 4 weeks by FACS.

(F) Bar graphs showing the populations of LSK cells, and LT-HSCs in the BM of recipient mice with C-176 treatment analyzed by FACS.

(G) C-176 treatment promotes erythropoiesis and inhibits skewed myelopoiesis of *Tet2^-/-,Vav^* donor BM cells in cBMT assays (1 donor and 8 recipients). Mice were treated with DMSO or C-176 by i.p. every 3 days and sacrificed after 8 weeks of inhibitor treatment. The erythropoiesis and myelopoiesis of recipients were analyzed by FACS.

Statistical significance was assessed with two-way ANOVA (B, E) or t-test (C, D, F, G). Data are mean ± s.e.m., **P* < 0.05; ***P* < 0.01; ****P* < 0.005; “ns”: not significant.

**Supplementary figure 6. cGAS-STING pathway is activated in TET2-knockdown human HSCs**

(A) *TET2* and *IFNβ* expression in TET2-knockdown CD34^+^ cells quantified by qRT-PCR (2 different shRNA constructs, 3 biological replicates). CD34^+^ cells were isolated from cord blood and transduced with sh*TET2* or sh*Ctrl* lentivirus. The expression levels of *TET2* and *IFNβ* were analyzed 72h post transduction.

(B) The number of micronuclei in binucleated cells is increased in TET2-knockdown CD34^+^ cells. To ensure that cells only underwent one division, CD34^+^ cells were treated with Cytochalasin B. The left panel shows binucleated cells with and without micronuclei, and the quantification is shown on the right (n = 3 biological replicates).

(C) Immunofluorescence showing cGAS (red signals) colocalizing with micronucleus in TET2-knockdown CD34^+^ cells. The arrow points to the micronucleus (Scale bar, 20 μm).

Statistical significance was assessed by t-test (A, B). Data are mean ± s.e.m. **P* < 0.05.

**Supplementary figure 7. Activated cGAS-STING pathway mediates skewed myeloid differentiation of TET2-knockdown human HSCs in vitro.**

(A) STING depletion reduces the expression of *IFNβ* in TET2-knockdown CD34^+^ cells. The mRNA levels of *TET2*, *STING,* and *IFNβ* were quantified by qRT-PCR (2 different shRNA constructs for each gene, n = 3 biological replicates).

(B) STING depletion attenuates myeloid colony formation and restores erythroid colony formation of TET2-ablated CD34^+^ cells (1 shRNA construct for TET2 and 2 different shRNA constructs for STING, n = 3 biological replicates).

Statistical significance was assessed by t-test (A, B). Data are mean ± s.e.m. **P* < 0.05; “ns”: not significant.

**Supplementary figure 8. STING inhibition ameliorates the leukemogenesis of TET2-mutated mononuclear cells in AML patients-derived xenograft models.**

(A) AML patient information.

(B) *TET2* expression is downregulated in TET2-mutated AML patient cells and corelated with the upregulation of inflammatory cytokines *IFNβ* and *IL6*. The mRNA levels of *TET2, IFNβ* and *IL6* were quantified by qRT-PCR (n = 3 biological replicates).

(C) STING inhibitor H-151 has no effect on the engraftment of TET2-WT patient AML cells in the transplantation assay. Recipient-mice were treated with DMSO or H-151 through i.p. every 3 days and the percentages of human CD45^+^ cells in the peripheral blood were analyzed 14 weeks after transplantation (n = 3 mice).

(D) H-151 reduces the expansion of TET2-mutated patient AML cells in the peripheral blood of B-NDG mice (n = 3 mice). Mice were treated with DMSO or H-151 through i.p. every 3 days and the engraftments of human CD45^+^ cells in the peripheral blood were analyzed by FACS every 6 weeks. All three recipient mice of P7 died at week 7.

Statistical significance was assessed with two-way ANOVA (B) and t-test (C, D). Data are mean ± s.e.m. **P* < 0.05, ***P* < 0.01, ****P* < 0.005. “ns”: not significant.

**Supplementary methods**

**Lentivirus preparation**

All shRNAs were expressed in pLKO.1-copGFP or pLKO.1-BFP lentivirus with the target sequences as follows, human *STING*-1 (GCATCAAGGATCGGGTTTACA), *STING*-2 (GGATCGGGTTTACAGCAAC), *TET2*-1 (CCATCACAATTGCTTCTTT), *TET2*-2 (ACACCCTCTCAAGATTGTTTA); mouse *Sting* (GCATCAAGAATCGGGTTTATT), mouse *Mavs* (CCAGTGCTGATCTATTAGGAA), A pLKO.1 scramble control plasmid containing the following target sequence was used: CCTAAGGTTAAGTCGCCCTCG^1^. Lentiviral production was performed as previously described^2^.

**Colony formation assay**

Lineage^-^cKit^+^Sca1^+^ (LSK) cells were isolated from the BM cells of transgenic mice and plated into a 24-well plate with methylcellulose medium (M3434; StemCell Technologies) at300 cells per well. Colony forming units were replated (1000 cells per well) every 7 days and colonies were scored at day 7 post-plating. To test the effect of C-178 and IFNβ, LSK cells were cultured in the presence of 0.5 μM C-178 and/or 0.2 unit/ml IFNβ. Human cord blood CD34^+^ cells isolated through FACS were plated in methylcellulose medium (H4434, StemCell Technologies) at 600 cells per well and colonies were scored two weeks after plating. Mononuclear cell (MNC) fractions were obtained from BM of patients with AML by density gradient centrifugation using Ficoll-Paque Plus (GE Healthcare). MNCs were plated into methylcellulose medium (H4434, StemCell Technologies) at 1 × 10^4^ cells per well in the presence of DMSO or 1 μM H-151. Plates were incubated at 37 °C at 5% CO_2_, and colonies were counted two weeks after plating.

**HSC** **limiting dilution assay**

Bone marrow cells were collected from mice of different genotypes (CD45.2) and diluted to a concentration of 1×10^5^, 3×10^4^, 1.5×10^4^, 6×10^3^, and 3×10^3^. These cells were mixed with 3×10^5^ bone marrow cells from B6.SJL mice (CD45.1) and injected into lethally irradiated (9.5Gy) recipient mice (CD45.1) aged 8-10 weeks. For the first two doses of cell mixtures, each dose contains three recipients, while for the other doses, each dose contains six recipients. The populations of CD45.2^+^ cells were measured every four weeks after transplantation. Sixteen weeks after transplantation, the reconstitution efficiency (CD45.2^+^ > 1%) of each donor mouse genotype was calculated using L-Calc (StemCell) software.

**Transduction of human cord blood CD34^+^ cells and mouse cKit^+^ cells**

Human cord blood CD34^+^ cells were enriched using a magnetic bead sorting system according to the manufacturer’s instructions (Miltenyi Biotec, Bergisch Gladbach, Germany). For the initial expansion, cord blood CD34^+^ cells were cultivated in StemSpan SFEM (09650, StemCell Technologies) supplemented with 100 ng/ml human stem cell factor (SCF), 50 ng/ml Fms-like tyrosine kinase 3 ligand (Flt-3L) and 50 ng/ml thrombopoietin (TPO) for 1 day. The pre-stimulated CD34^+^ cells were infected with lentivirus carrying shRNA targeting *TET2*, *STING* or a control vector in the presence of polybrene (5 μg/ml, Sigma). The positive cells were sorted on the flow cytometer (FACS Aria III, BD Biosciences, San Jose, CA) 72 hours post-infection. For colony-forming assays, the sorted GFP^+^BFP^+^ cells were cultured in methylcellulose medium (H4434, StemCell Technologies) supplemented with 10% IMDM (Sigma) basic culture medium for 14 days.

Mouse BM LSK cells harvested from transgenic mice were cultured overnight in StemSpan SFEM supplemented with mouse recombinant SCF (100 ng/ml), IL-6 (10 ng/ml) and IL-3 (10ng/ml). The next day, cells were infected with lentivirus carrying shRNA targeting *Sting, Mavs* or an empty vector control in the presence of polybrene (5 μg/ml, Sigma) and centrifuged at 1000g, 32℃ for 1 hour. The spin infection was repeated the next day. Forty-eight hours after transduction, the GFP positive cells were sorted, and 1 × 10^5^ cells were lysed to determine the knockdown efficiency and cytokine expression.

**Flow cytometric analysis and cell sorting**

Single-cell suspensions from BM and PB were stained with fluorochrome-conjugated antibodies. For lineage stratification, cells from PB and BM were stained with FITC-Gr1, PE-cy7-Mac1, FITC-CD4, PE-cy7-CD8, PE-Ter119 and APC-CD71 for 30min at 4°C. For MPP2/3/4 cells, LT-HSCs and ST-HSCs, BM cells were stained with FITC-conjugated lineage cocktail (B220, CD4, CD5, CD8, Gr1 and Ter119), Percp-cy5.5-Sca1, BV421-cKit, PE-CD150, APC-cy7-CD48, APC-Flk2. LT-HSC were immunophenotypically defined as Lin^-^cKit^+^Sca1^+^CD48^-^CD150^+^, ST-HSCs as Lin^-^cKit^+^Sca1^+^CD48^-^CD150^-^, MPP2 as Lin^-^cKit^+^Sca1^+^Flk^-^CD48^+^CD150^+^, MPP3 as Lin^-^cKit^+^Sca1^+^Flk^-^CD48^+^CD150^-^, MPP4 as Lin^-^cKit^+^Sca1^+^Flk^+^CD48^+^CD150^-^. For committed progenitor cells, granulocyte-monocyte progenitors (GMP) were immunophenotypically defined as Lin^-^cKit^+^ Sca1^-^CD34^+^CD16/32^high^, common myeloid progenitors (CMP) as Lin^-^cKit^+^Sca1^-^CD34^int/+^CD16/32^int^, megakaryocyte-erythrocyte progenitors (MEP) as Lin^-^cKit^+^Sca1^-^CD34^-^CD16/32^low^) and common lymphoid progenitors (CLP) as lin^-^cKit^+^Sca1^int^ CD127^+^. The analyses and sorting were performed using a BD FACScanto II cytometer or a BD FACSAria™ III cell sorter. All data were analyzed using FlowJo software, version 10.

**Immunofluorescence assay**

FACS-sorted mice hematopoietic cells and human cord blood CD34^+^ cells were concentrated using a cytospins for immunofluorescence analysis. Immunofluorescence was performed as previously described^5^. The following primary antibodies were applied: anti-phospho-H2AX (Ser139) (Millipore, 05-636), anti-cGAS (CST 79978). Images were acquired using a Leica TCS SP8 confocal laser microscopy system.

**PCR and Real-time quantitative PCR**

PCR Genotyping primers were as follows: *Tet2*: 5’-ACTCATTAGTGAAATATGTGAGTG-3’, 5’-CTGCTTAGTTCAATGCCAACC-3’, 5’-ACACAGAGAAAAGGGTACGTGAA-3’. Wild-type band = 447 bp, floxed band = 536 bp, knockout band = 617 bp. *Sting*: 5’-TGCTGTAGGATGCTATGTGC-3’, 5’-ACAGAGGGTTACCTGGACTG-3’ and confirmed by PCR product sequencing.

Total RNA was extracted with TRIzol Reagent (Invitrogen, Thermo Fisher Scientific), and cDNA was synthesized using HyperScript III 1st Strand cDNA Synthesis Kit with gDNA Remover (EnzyArtisan, R201) according to the manufacturer’s instructions. Real-time quantitative PCR was performed with S6 Universal SYBR qPCR Mix (EnzyArtisan, Q204) on a LightCycler 480 II (Roche Applied Science). Expression of genes of interest were normalized to the housekeeping gene *Actb* or *GAPDH* using the 2–ΔΔCt method. qPCR primers were as follows, mouse *Ifnβ*: 5‘-CCCTATGGAGATGACGGAGA-3’, 5’-CTGTCTGCTGGTGGAGTTCA-3’. Mouse *Il-6*: 5’-TCCATCCAGTTGCCTTCTTG-3’, 5’-GGTCTGTTGGGAGTGGTATC-3’. Mouse *Actb*: 5’-TGACGTTGACATCCGTAAAGACC-3’, 5’-AAGGGTGTAAAACGCAGCTCA-3’. Mouse *Sting*: 5’-GTCTAGGAAGCAGAAGATGCCA-3’, 5’- GAGGACCAGAAGGCCAAACA-3’. Mouse *Mavs*: 5’-CTGCCTCACAGCTAGTGACC-3’, CCGGCGCTGGAGATTATTG. Human *TET2*: 5’-GGAAAGCTTTTCAGCTGCAGC-3’, 5’-CTTGCACAACATGCAGAATGGCAGC-3’. Human *GAPDH*: 5’-AGAAGGCTGGGGCTCATTTG-3’, 5’-AGGGGCCATCCACAGTCTTC-3’. Human *IFNβ*: 5’- CAGCAGTTCCAGAAGGAGGA-3’, 5’-AGCCAGGAGGTTCTCAACAA-3’. Human *STING*: 5’-CCTTGGTTCTGCTGAGTGCC-3’, 5’-CCGGTACCTGGAGTGGATGT-3’.

**RNA-seq library preparation**

About 300 hematopoietic cells per group were sorted directly into Smart-seq2 lysis buffer by FACS. Sorted cells were lysed and the reverse-transcribed RNA was amplified to obtain enough cDNA by a modified SMART-Seq2 protocol^3,4^. cDNA was quantified by Qubit 3 (Invitrogen, Q33327), and 5 ng cDNA was used for cDNA library construction with TruePrep DNA Library Prep Kit V2 for Illumina (Vazyme, Cat. TD502).

**RNA-seq data analysis**

The raw pair-end RNA-seq FASTQ data were trimmed to remove low-quality bases and adaptor sequences by Trim Galore (v0.5.0) with default settings. Then, the clean RNA-seq FASTQ data were mapped to mouse reference genome mm10 using Hisat2 (v2.1.0) with default parameters. Differentially expressed gene (DEG) analysis was performed by using DESeq2 R package with the raw count. Only genes with adjusted P value less than 0.05 and at least 2-fold-change were considered to be differentially expressed. DEGs were submitted to DAVID 6.7 (https://david.ncifcrf.gov) for gene ontology (GO) and KEGG pathway enrichment analyses.

**Western Blot Analysis**

Equal numbers of cells from each population were isolated with FACS or LS column (Miltenyi Biotec. Cat. No. 130-042-401). Cells were collected and resuspended in lysis buffer containing Tris 8.0 20mM, NaCl 150mM, Triton X-100 0.5%, protease inhibitor cocktail (Roche). Lysates were subjected to ultrasonication for 5 min to disrupt the genomic DNA and then mixed with SDS loading buffer. Blots were developed with ECL detection reagent (180-5001, Tanon) and imaged on a CCD imager (GE Healthcare).

**Human subjects**

Diagnostic bone marrow was obtained from patients with AML treated at the Huashan Hospital of Fudan University. All cells were collected after obtaining informed consent from AML patients and the study followed the ethical guidelines of Huashan Hospital of Fudan University. Human CD34^+^ cells were isolated from cord blood of healthy donors after obtaining informed consent.

**Patient-derived xenografts**

3 × 10^6^ mononuclear cells (MNCs) from AML patients were transplanted into sub-lethally irradiated (1.8Gy) B-NDG mice. When the average engraftment of hCD45 cells in PB reached 1%, mice were treated with either DMSO (corn oil) or 2 μmol/body H-151 every other day for 30 days. %hCD45 engraftment in PB was analyzed after 5 doses, and mice were sacrificed at day 30 to assess the leukemia burden in BM.

**Generation of ^13^C_10_^15^N_5_-labeled cGAMP**

Recombinant His-SUMO-cGAS (human) was expressed and purified in *E. coli* strain Rosetta (DE3) as previously described^5^. For ^13^C_10_^15^N_5_-labeled cGAMP in vitro synthesis, 100 μg of His-SUMO-cGAS protein was mixed with Buffer R (20 mM HEPES, pH 7.2, 5 mM MgCl_2_, 1 mM ATP-^13^C_10_,^15^N_5_, 1 mM GTP, and 0.1 mM EGTA) in the presence of 0.1 mg/ml HT-DNA. The mixture was incubated at 37°C for 1 hour, then heated at 95°C for 5 min, centrifuged at 10000g, 4℃, for 10 min. The ^13^C_10_^15^N_5_-labeled cGAMP was in the heat-resistant supernatant and was used as an internal standard for the quantification of cellular cGAMP.

**Extraction of endogenous cGAMP in BM-MNCs**

Total BM-MNCs (1 mouse) were collected and washed twice with cold PBS, resuspended in cold 80% (vol/vol) methanol with 2% (vol/vol) acetic acid (HAc), and stored at −80 °C. On the day of analysis, 50 fmol ^13^C_10_^15^N_5_-labeled cGAMP (+15 atomic mass units) was added into the BM cells as internal standard. Cells were homogenized for 20 s and centrifugated at 12000g for 10 min. The pellets were extracted in 20% (vol/vol) methanol and 2% HAc, and all supernatants were pooled. The supernatants were then loaded onto HyperSep Aminopropyl SPE Columns (Thermo Scientific) to remove impurities and enrich cGAMP. Briefly, the columns were washed sequentially with 1 ml methanol and 500 μl 2% HAc for two times; then, all supernatants were loaded onto SPE columns (about 1ml); after drawing through the extracts, columns were washed twice with 500μl 2% HAc and once with 500μl 80% methanol; cGAMP enrichments were eluted with 4% (vol/vol) ammonium hydroxide in 80% methanol. The eluents were spin-vacuumed to dry and reconstituted in 40% acetonitrile: 40% methanol: 20% H_2_O (vol/vol). The eluents were cleared by centrifugation and transferred to autosampler vials for MS analyses.

**Quantification of cGAMP with LC-MS**

The LC-MS/MS analysis was performed on an Agilent 1290 Infinity Ⅱ LC System equipped with a ACQUITY UPLC BEH Amide column (1.7 µm, 2.1 × 150 mm; Waters) coupled by ESI to a QTRAP 6500^+^ (AB Sciex). The column was maintained at 35°C and eluents were injected at a constant flow rate of 300 µl/min. The binary mobile phase was composed of 0.125% formic acid in 50/50 (vol/vol) acetonitrile/water (A) and 90/10 (vol/vol) acetonitrile/water (B), and samples were flowed through the following gradient: 0 min, 99% B; 3 min, 99% B; 5 min, 50% B; 5.5 min, 35% B; 10.5 min, 30% B; 11 min, 5% B; 14.5min, 5% B; 15 min, 99% B, and 20 min, 99% B. The mass spectrometer was operated in the positive ion mode with the following settings: ion spray voltage: +5500 V, declustering potential: 85 V, entrance potential: 10 V, collision energy: 32 V, source temperature: 550°C, and curtain, ion source gas at 35, 55. cGAMP and the ^13^C_10_^15^N_5_-labeled cGAMP were detected in multiple reaction monitoring (MRM) mode for four mass transitions, respectively (cGAMP: 675-136, 675-152, 675-476, and 675-524; and the ^13^C_10_^15^N_5_-labeled cGAMP: 691-146, 691-152, 691-491, and 691-539). Peak identities were verified by the analysis of commercial cGAMP and ^13^C_10_^15^N_5_-labeled cGAMP spike-in and selected areas were integrated with the Sciex OS software 2.0. Relative abundance of endogenous cGAMP was calculated based on the difference of peak aeras between the control and *Tet2*-deficient groups using a calibration curve, and was normalized to internal standard.

**Comet assay**

Mouse BM LSK cells were collected and resuspended in phosphate-buffered saline (PBS) at 1 × 10^5^ cells per ml. The comet assay was performed according to the manufacturer’s instructions (4250-050-K; Trevigen, Gaithersburg, Maryland). DNA damage was measured by tail moments using comet-score software.

**Statistical analysis**

Data are shown as mean ± s.e.m. To assess the statistical significance, we used unpaired Student’s t-tests for comparison between two groups and two-way (more than two genotypes) ANOVA for multiple groups. Survival curves were compared using Mantel-Cox log-rank test. All statistical tests were performed using GraphPad Prism software.

**Supplementary References**

1 Sarbassov, D. D., Guertin, D. A., Ali, S. M. & Sabatini, D. M. Phosphorylation and Regulation of Akt/PKB by the Rictor-mTOR Complex. **307**, 1098-1101, doi:doi:10.1126/science.1106148 (2005).

2 Ran, F. A. *et al.* Genome engineering using the CRISPR-Cas9 system. *Nature Protocols* **8**, 2281-2308, doi:10.1038/nprot.2013.143 (2013).

3 Ramsköld, D. *et al.* Full-length mRNA-Seq from single-cell levels of RNA and individual circulating tumor cells. *Nature Biotechnology* **30**, 777-782, doi:10.1038/nbt.2282 (2012).

4 Xu, Q. *et al.* Loss of TET reprograms Wnt signaling through impaired demethylation to promote lung cancer development. **119**, e2107599119, doi:doi:10.1073/pnas.2107599119 (2022).

5 Sun, L., Wu, J., Du, F., Chen, X. & Chen, Z. J. Cyclic GMP-AMP Synthase Is a Cytosolic DNA Sensor That Activates the Type I Interferon Pathway. *Science* **339**, 786-791, doi:doi:10.1126/science.1232458 (2013).
