# Supplementary figures and images for: STING activation in TET2-mutated hematopoietic stem/progenitor cells contributes to the increased self-renewal and neoplastic transformation
Source: Leukemia. 2023 Oct 10;37(12):2457–67. doi: 10.1038/s41375-023-02055-z (PMC10681905; doi:10.1038/s41375-023-02055-z)

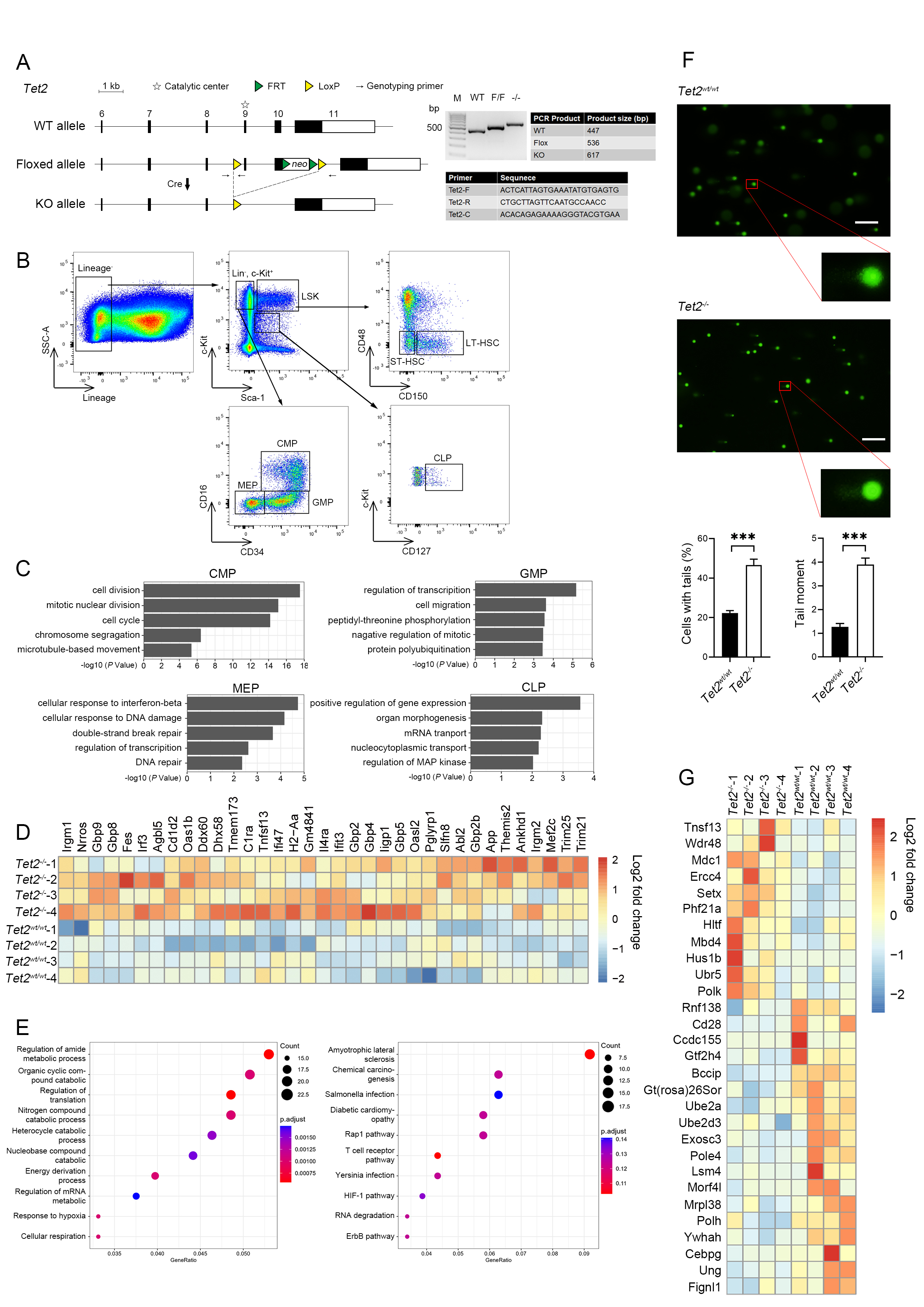

Supplement: Supplementary file 2 — Supplementary Figure 1 [file 41375_2023_2055_MOESM2_ESM.jpg]

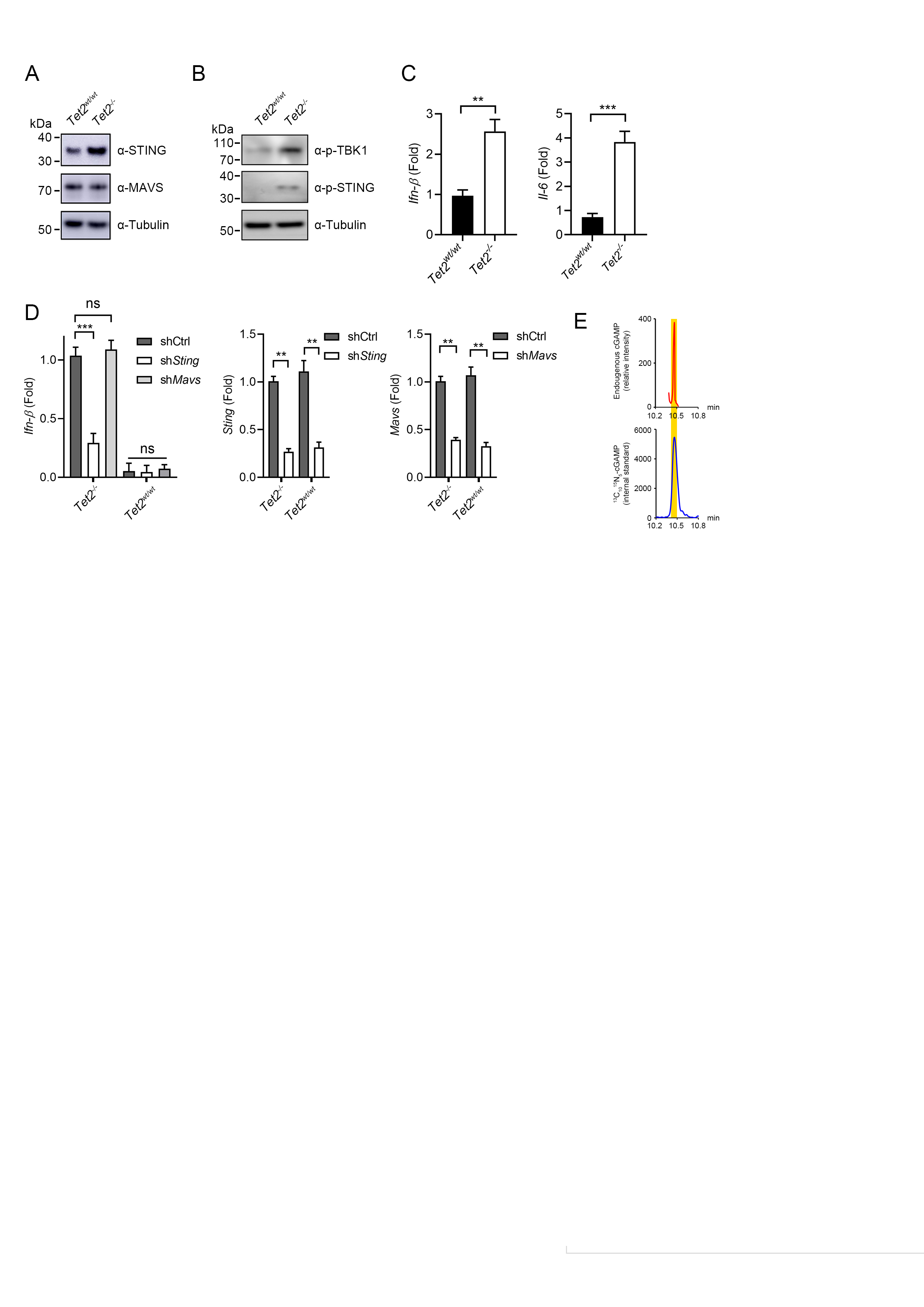

Supplement: Supplementary file 3 — Supplementary Figure 2 [file 41375_2023_2055_MOESM3_ESM.jpg]

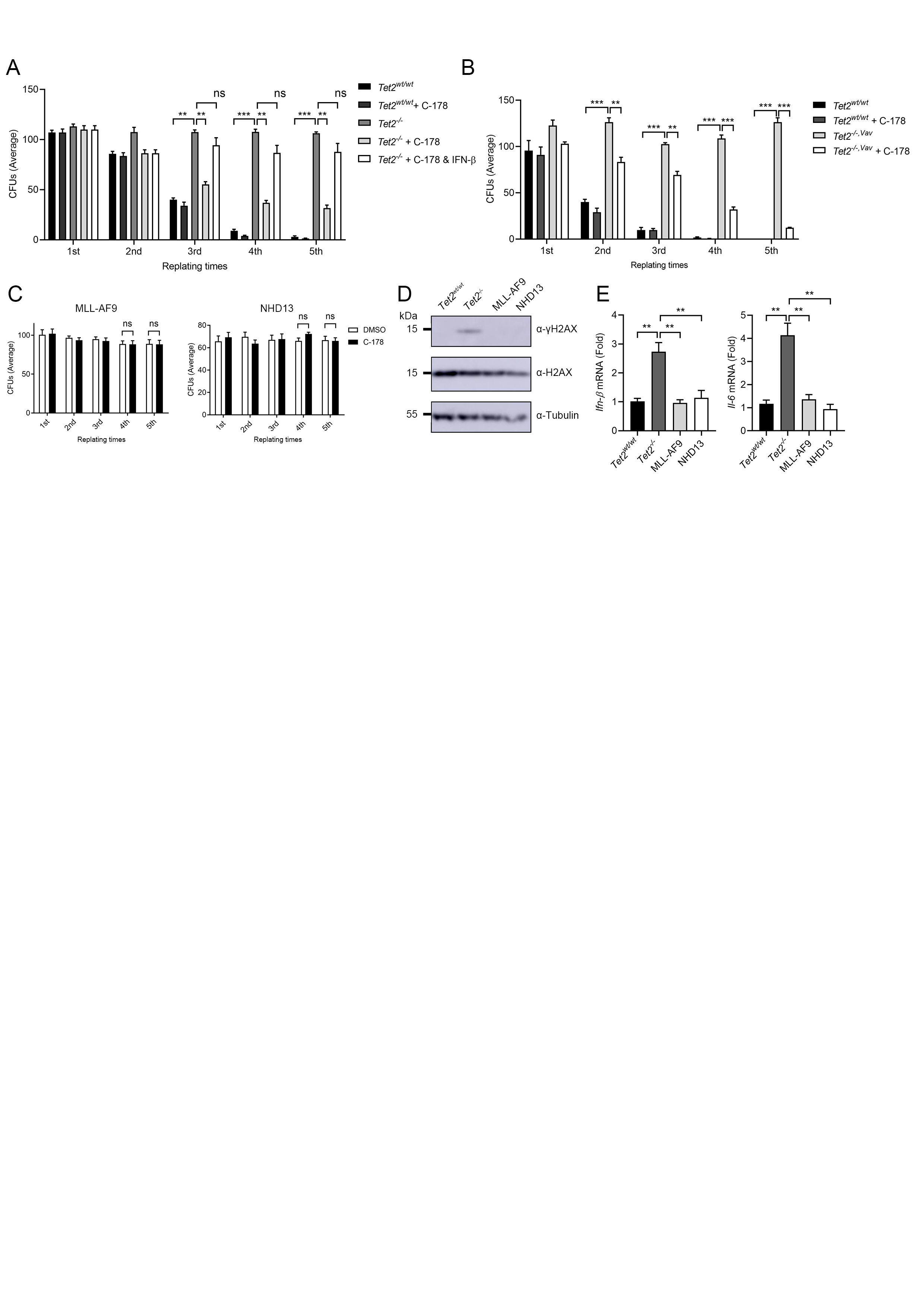

Supplement: Supplementary file 4 — Supplementary Figure 3 [file 41375_2023_2055_MOESM4_ESM.jpg]

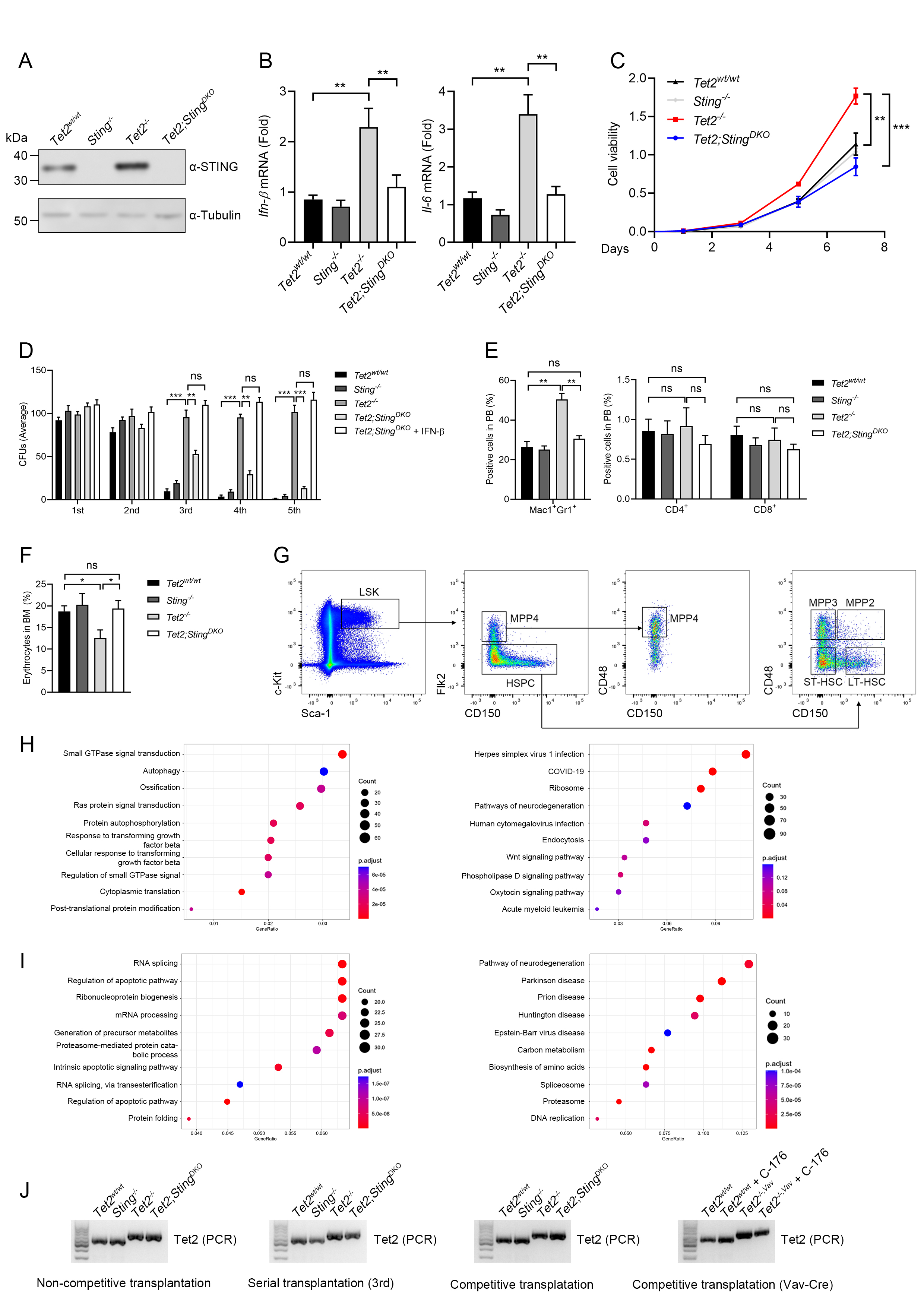

Supplement: Supplementary file 5 — Supplementary Figure 4 [file 41375_2023_2055_MOESM5_ESM.jpg]

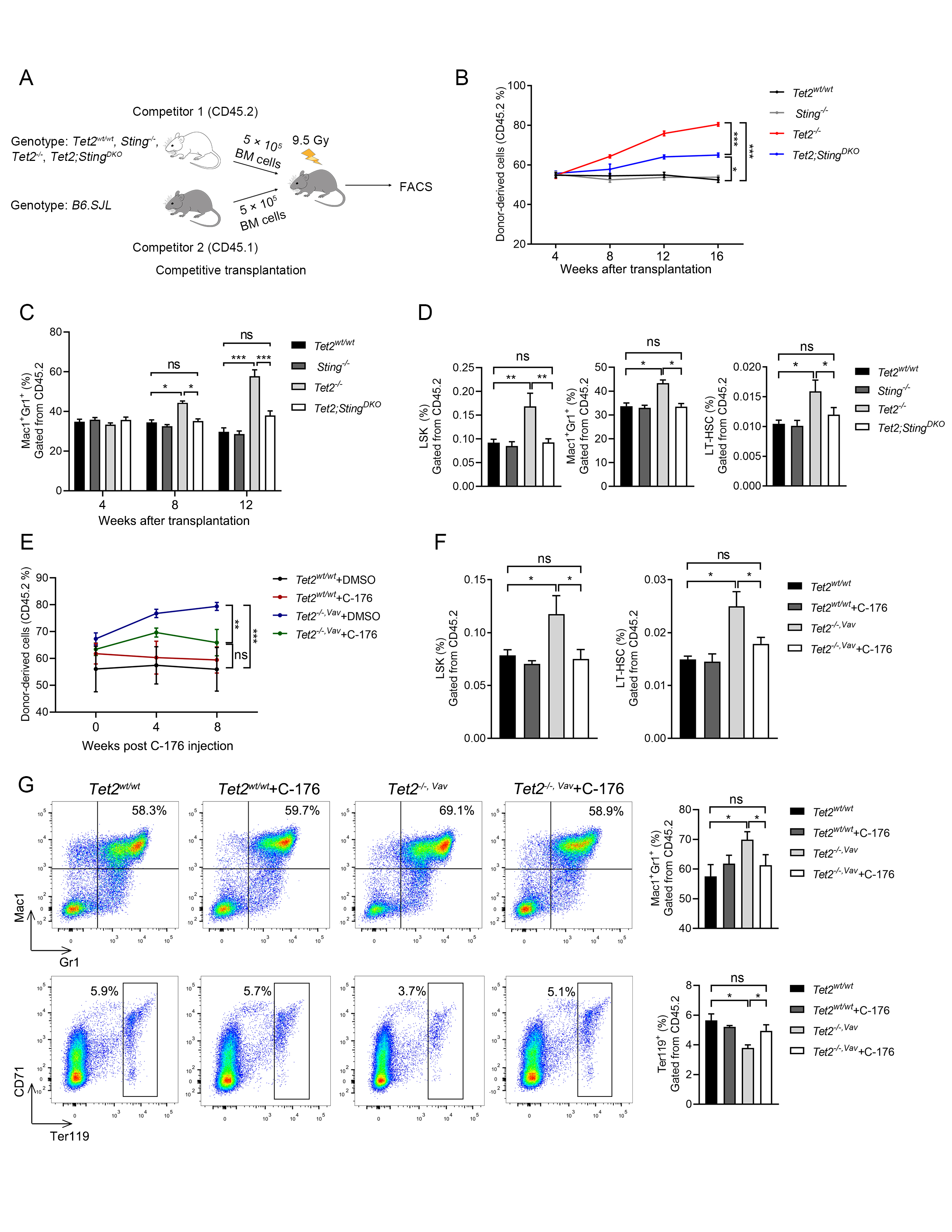

Supplement: Supplementary file 6 — Supplementary Figure 5 [file 41375_2023_2055_MOESM6_ESM.jpg]

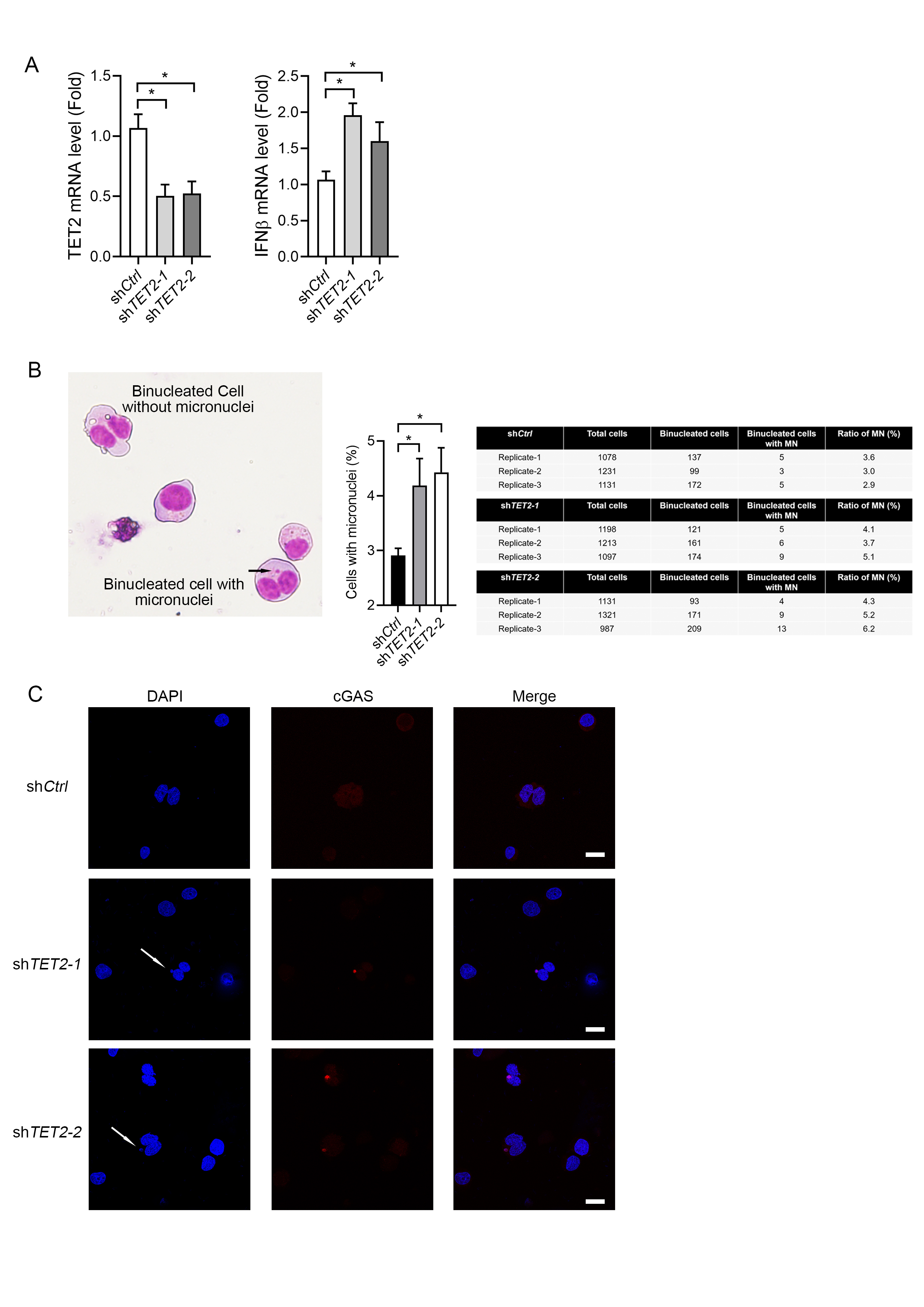

Supplement: Supplementary file 7 — Supplementary Figure 6 [file 41375_2023_2055_MOESM7_ESM.jpg]

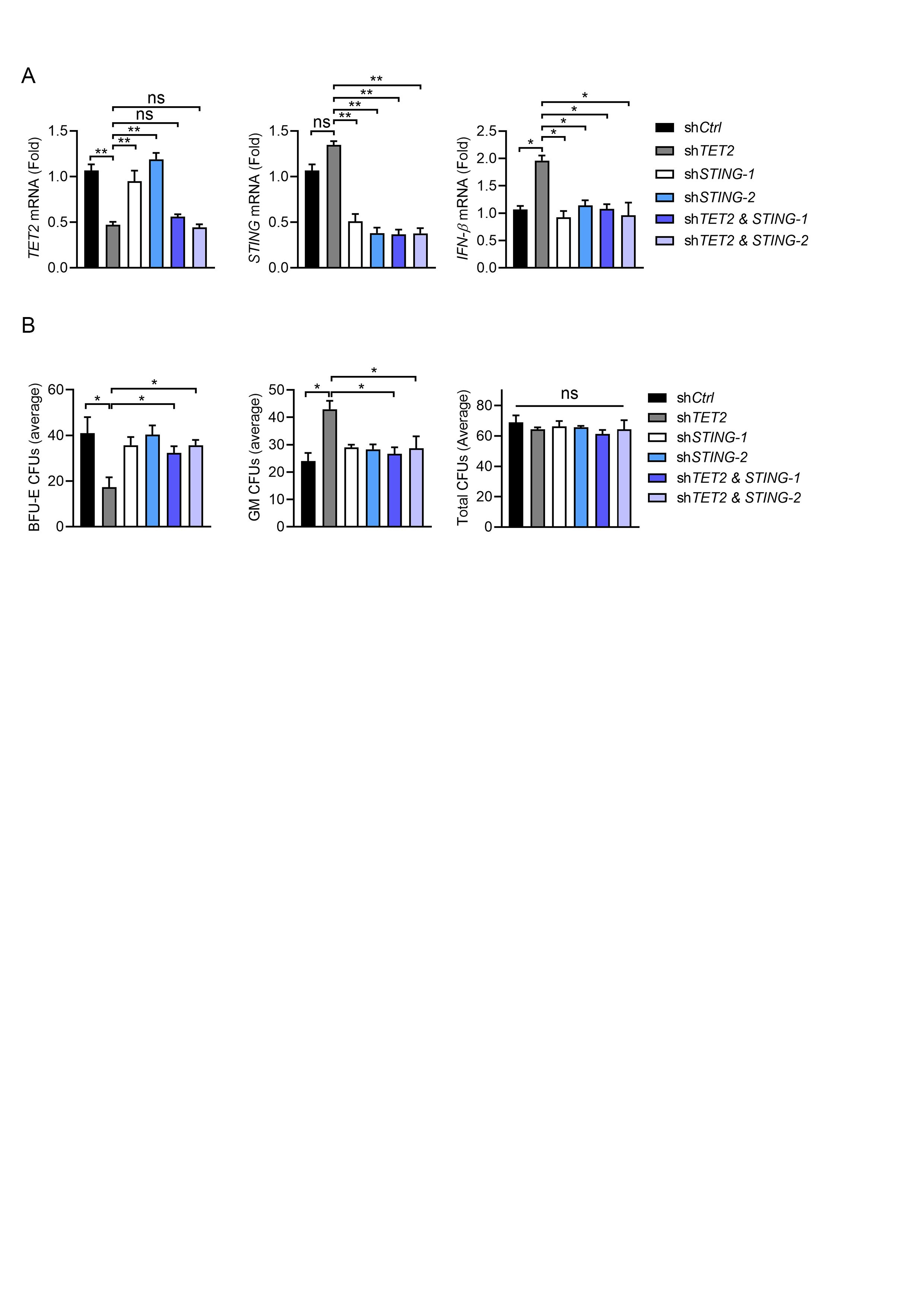

Supplement: Supplementary file 8 — Supplementary Figure 7 [file 41375_2023_2055_MOESM8_ESM.jpg]

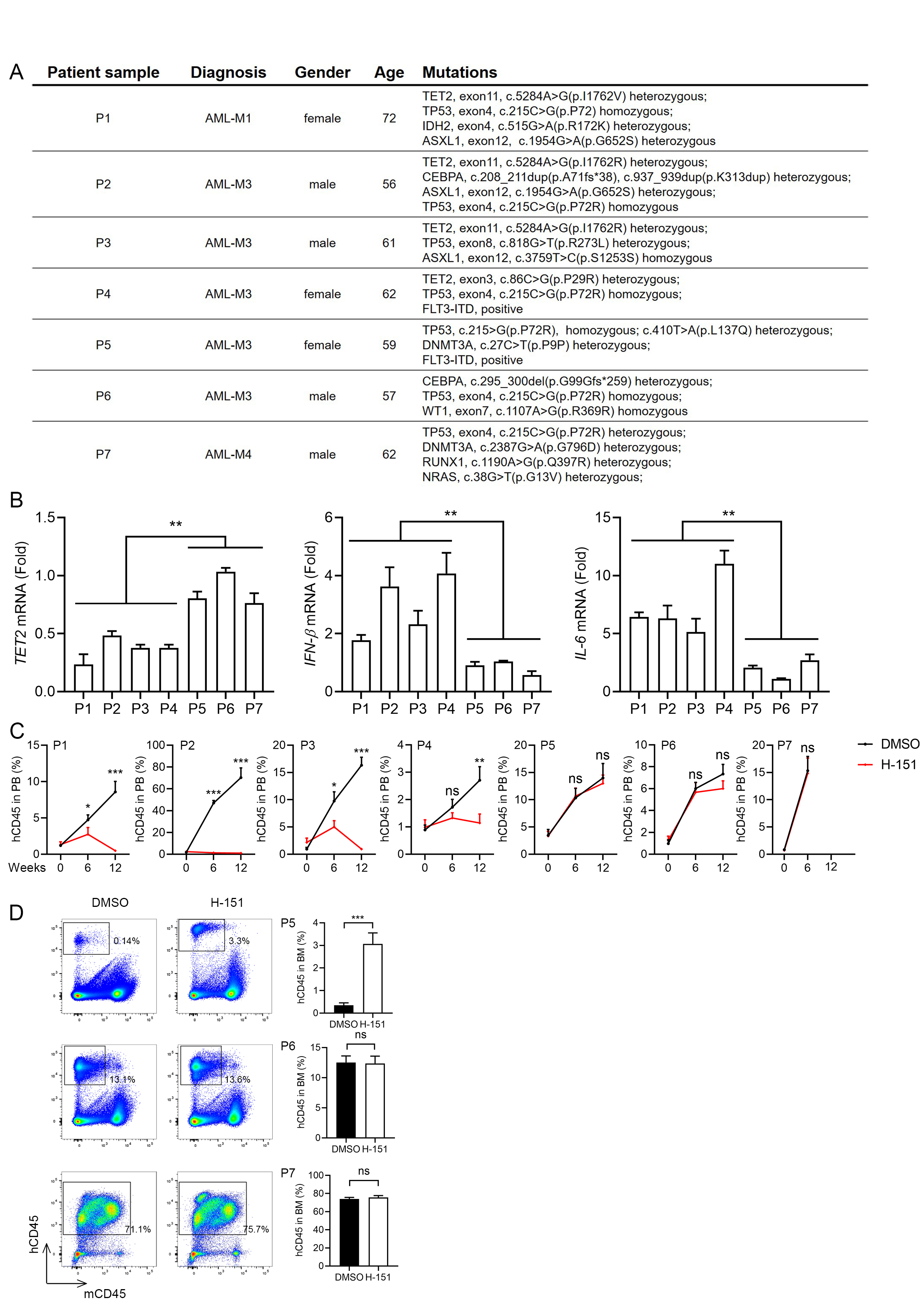

Supplement: Supplementary file 9 — Supplementary Figure 8 [file 41375_2023_2055_MOESM9_ESM.jpg]
